# Creation of a novel telomere-cutting endonuclease based on the EN domain of telomere-specific non-long terminal repeat retrotransposon, TRAS1

**DOI:** 10.1186/1759-8753-1-13

**Published:** 2010-04-01

**Authors:** Kazutoshi Yoshitake, Hideyuki Aoyagi, Haruhiko Fujiwara

**Affiliations:** 1 Department of Integrated Biosciences, Graduate School of Frontier Sciences, The University of Tokyo, Bioscience Bldg 501, Kashiwa, 277-8562, Japan

## Abstract

**Background:**

The ends of chromosomes, termed telomeres consist of repetitive DNA. The telomeric sequences shorten with cell division and, when telomeres are critically abbreviated, cells stop proliferating. However, in cancer cells, by the expression of telomerase which elongates telomeres, the cells can continue proliferating. Many approaches for telomere shortening have been pursued in the past, but to our knowledge, cutting telomeres *in vivo *has not so far been demonstrated. In addition, there is lack of information on the cellular effects of telomere shortening in human cells.

**Results:**

Here, we created novel chimeric endonucleases to cut telomeres by fusing the endonuclease domain (TRAS1EN) of the silkworm's telomere specific non-long terminal repeat retrotransposon TRAS1 to the human telomere-binding protein, TRF1. An *in vitro *assay demonstrated that the TRAS1EN-TRF1 chimeric endonucleases (T-EN and EN-T) cut the human (TTAGGG)_n _repeats specifically. The concentration of TRAS1EN-TRF1 chimeric endonucleases necessary for the cleavage of (TTAGGG)_n _repeats was about 40-fold lower than that of TRAS1EN alone. When TRAS1EN-TRF1 endonucleases were introduced into human U2OS cancer cells using adenovirus vectors, the enzymes localized at telomeres of nuclei, cleaved and shortened the telomeric DNA by double-strand breaks. When human U2OS and HFL-1 fibroblast cells were infected with EN-T recombinant adenovirus, their cellular proliferation was suppressed for about 2 weeks after infection. In contrast, the TRAS1EN mutant (H258A) chimeric endonuclease fused with TRF1 (ENmut-T) did not show the suppression effect. The EN-T recombinant adenovirus induced telomere shortening in U2OS cells, activated the p53-dependent pathway and caused the senescence associated cellular responses, while the ENmut-T construct did not show such effects.

**Conclusions:**

A novel TRAS1EN-TRF1 chimeric endonuclease (EN-T) cuts the human telomeric repeats (TTAGGG)_n _specifically *in vitro *and *in vivo*. Thus, the chimeric endonuclease which is expressed from an adenoviral vector can suppress cell proliferation of cancer cells.

## Background

Telomeres are specialized structures that protect chromosomal ends from gene erosion at cell divisions, nonhomologous chromosomal end joining and nuclease attack [1]. The DNA component of telomeres, typically 5-8 kb long, consists of tandem arrays of short, repetitive G-rich sequences--TTAGGG in vertebrates--oriented 5'- to -3' towards the end of the chromosome [2]. Each replication of human nuclear DNA results in a 50-200 base pair loss of the telomere. After reaching a critical shortening of the telomere, the cell enters replicative senescence or undergoes programmed cell death [3]. In striking contrast, the length of telomeres in cancer cells does not shorten during replication and remains constant over succeeding generations. This is because of the characteristic enzyme, telomerase, which is expressed in more than 80% of human cancers [4]. Expression of telomerase is not induced in normal human tissues and somatic cells and is usually limited to germline cells. Hence, telomerase is potentially a molecular target for developing antitumour agents [5]. However, as mentioned above, because telomeres shorten by 50-200 bases every cell division under physiological conditions, it takes a long time with conventional telomerase inhibitors (~1 month) [6] to induce effective telomere shortening followed by cellular senescence. In order to induce this rapidly, we investigated a novel method of shortening telomeres with the endonuclease domain of a telomere-specific non-long terminal repeat (LTR) retrotransposon, TRAS1.

Non-LTR retrotransposons, also known as long interspersed nuclear elements (LINEs), are transposable elements that insert into the genome via RNA intermediates. Most such transposable elements integrate into the genome randomly, although some LINEs have very restricted target sequences for integration [7]. TRAS1, found in the silkworm *Bombyx mori*, is a typical sequence-specific LINE, which inserts between the T and A of the (TTAGG)_n _telomeric repeats of the silkworm [8]. The second open reading frame (ORF2) of TRAS1 encodes an apurine/apyrimidine (AP) endonuclease-like domain (EN) at the *N*-terminal end [9]. The EN domain of TRAS1 (TRAS1EN) cuts the specific target sites of (TTAGG)_n_/(CCTAA)_n _telomeric repeats during target-primed reverse transcription, which is an essential process for LINEs [10]. TRAS1EN also cleaves the vertebrate telomeric repeats, (TTAGGG)_n _and several other telomere sequences in different species [11]. Thus, if TRAS1EN could cut telomeres in human cancer cells specifically and effectively, we hypothesized that the proliferation of cancer cells would be inhibited.

We have determined the crystal structure of TRAS1EN [11], which is basically similar to the AP endonuclease family, but has a special beta-hairpin at the DNA binding surface. Mutational studies of TRAS1EN indicated that the Asp-130 and β10-β11 hairpin structures are involved in specific recognition of telomeric repeats. We also analysed the structure of the EN of the silkworm 28S rDNA-specific LINE R1 (R1BmEN) and found that a Y98A mutant of R1BmEN on the DNA binding surface had altered cleavage patterns [12,13]. These results suggest an important role of the DNA binding surface in the EN of LINE for target sequence recognition and for the *de novo *design of a novel sequence-specific EN with altered sequence specificity for human telomeric repeats. Although *in silico *screening has been done for the homing endonuclease I-*Mso*I [14] to redesign its DNA binding and cleavage, it is still difficult to redesign *in silico *for changing the specificity of EN of LINE [15].

TRAS1EN cleaves human (TTAGGG)_n _telomeric repeats but its activity level and specificity seemed to be insufficient for practical application. In order to increase its ability, we used the human telomere-binding protein TRF1. The shelterin complex, consisting of six telomere-specific proteins including TRF1, protects the chromosomal ends. TRF1 has a Myb domain at its *C*-terminal end, which recognizes the double-strand DNA sequence TTAGGGTTA [16] and controls telomere length negatively [17]. We created chimeric endonucleases of TRAS1EN with either a full length of TRF1 or only the Myb domain of TRF1 and examined their abilities to cut vertebrate telomeric repeats. Here, we show that these TRAS1EN-TRF1 chimeric endonucleases cut TTAGGG repeats specifically *in vitro *and *in vivo*. They induce telomeric shortening and suppress cell proliferation when introduced into cancer cells.

## Results

### Construction and expression of chimeric endonucleases

**Figure 1:**
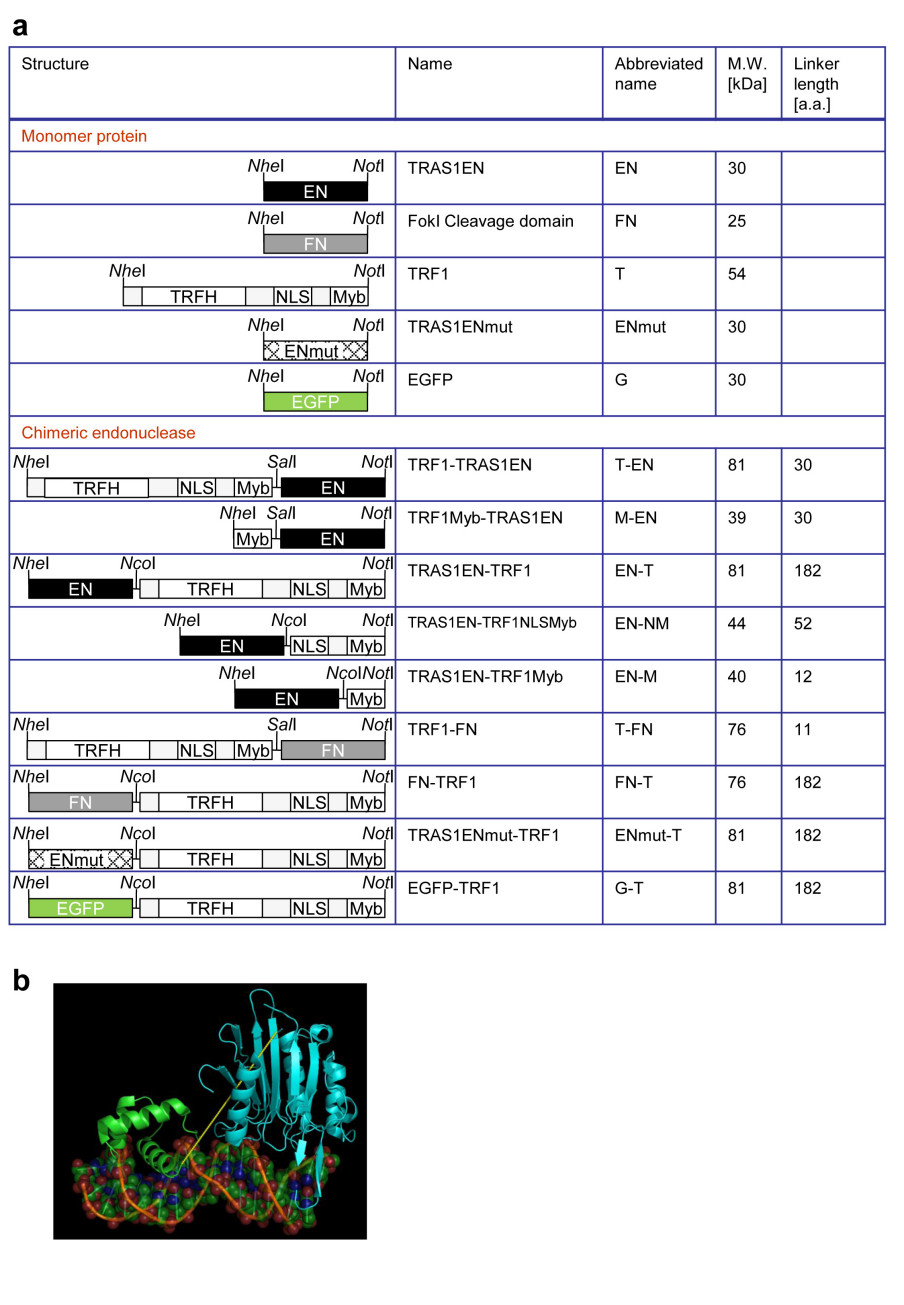
**Construction and structure of chimeric endonucleases**. (a) A schematic representation of fusion proteins constructed into pET21b for expression in *Escherichia. coli *is shown with the restriction sites. EN, TRAS1 endonuclease domain; FN, restriction endonuclease *Fok*I cleavage domain; TRF1, human telomere binding protein; TRFH, TRF heterodimerization region; NLS, nuclear localization signal; Myb, telomeric DNA binding motif. Estimated molecular weight and linker length are shown. (b) A model of the chimeric protein M-EN is shown with the *N*-terminal TRF1 Myb domain in green, the *C*-terminal TRAS1EN in cyan, and the interdomain hypothetical linker in yellow.

In order to increase the cleavage activity for human telomeric TTAGGG sequences, we constructed several types of chimeric endonuclease versions of TRAS1EN (EN), which cuts the silkworm TTAGG repeats by fusing wildtype TRAS1EN with various parts of TRF1 (T) either at the *N*- or *C*-terminal ends of TRAS1EN (Figure 1a). TRF1 has three domains: the homodimerization region (TRFH), a nuclear localization signal (NLS) and a telomeric DNA binding motif (Myb). Thus, we made chimeric EN constructs with a full-length sequence of TRF1 (T-EN, EN-T): only the TRF1-Myb component (M-EN, EN-M) and with the NLS to Myb region of TRF1 (EN-NM). As a negative control, we constructed a TRAS1EN mutant (ENmut, H258A) which has a mutation in the catalytic center that inactivates the endonuclease activity [9], and made respective chimeric constructs with TRF1 (T-ENmut, ENmut-T). We also used the nonspecific cleavage domain of a restriction enzyme *Fok*I (FN) which is often used for chimeric endonuclease design [18] and made FN constructs with a full length of TRF1 (T-FN, FN-T) (Figure 1a). We performed three-dimensional protein modelling in order to estimate each linker length of the chimeric constructs, based on the crystal protein structures. Figure 1b shows an example of the linker design (yellow line) in TRF1-Myb (M; green) and TRAS1EN (EN; cyan) of the chimeric endonuclease M-EN. The predicted structure of M-EN showed that the distance between M and EN was around 2-4 nm, indicating that the length of flexible linker between M and EN should be longer than 10 amino acids (assuming a peptide unit length of 3.8 Å), so that the EN domain can access target DNA effectively [19]. Similarly, we estimated and designed the linker for each chimera construct (Additional file 1). We expressed chimeric proteins with Rosetta2 *Escherichia coli *strain overexpressing tRNAs for rare codons and chaperons to enhance protein expression (Additional file 2a), analysed their qualities and quantities (Additional file 2b) and purified the proteins using the His tag.

### Chimeric endonucleases cleave human telomeric repeats effectively

**Figure 2:**
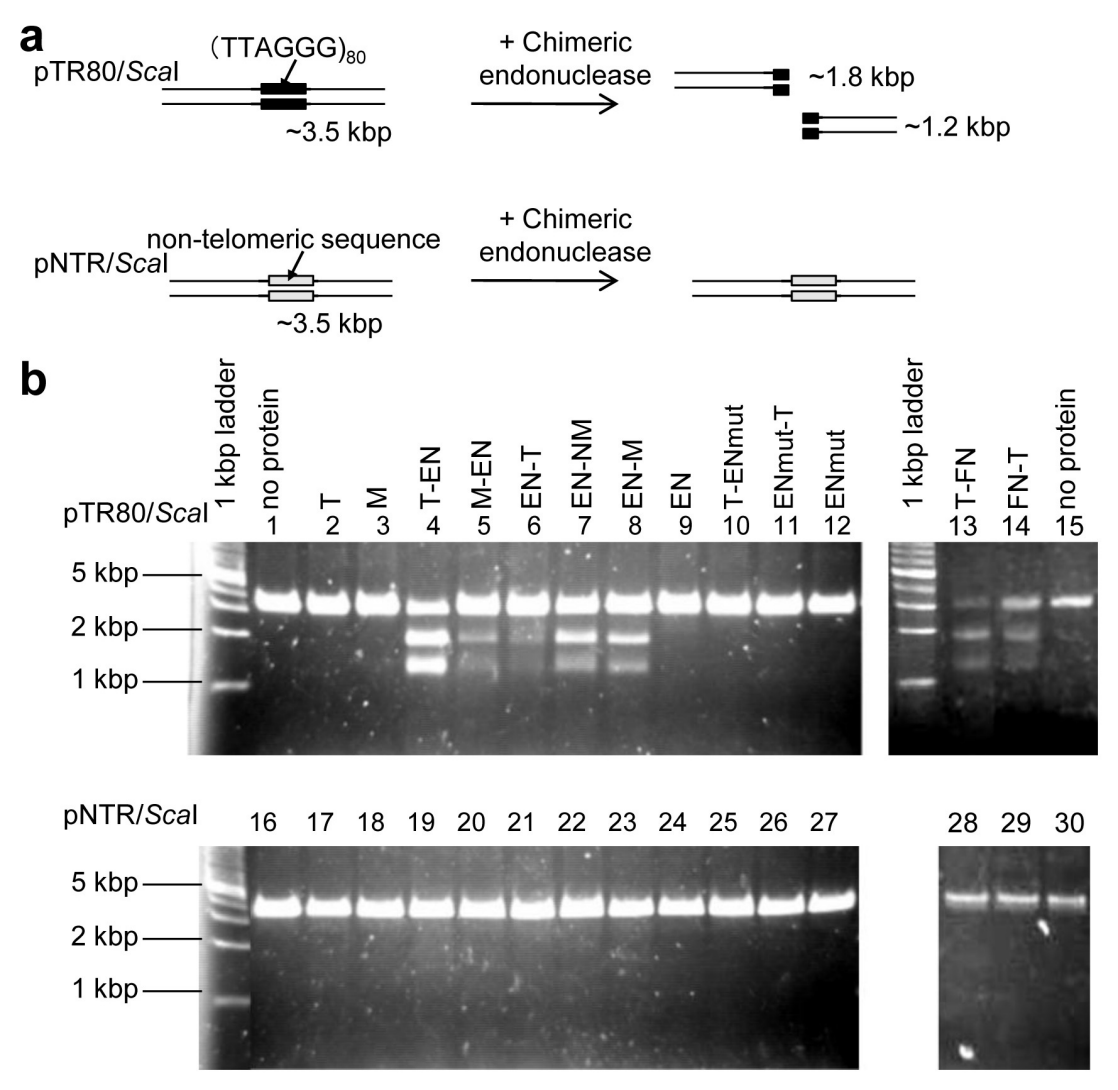
***In vitro *cleavage of human telomeric repeats by chimeric endonucleases**. (a) Schematic representation of cleavage assay. pTR80, 80 TTAGGG repeats were inserted in the TA cloning site of pGEM-T Easy vector. pNTR, a non-telomeric sequence inserted in the same vector (see Methods). (b) Digestion patterns with various chimeric endonucleases. Plasmids (36 ng) were added with about 0.3 μM purified proteins, except for FN-T (about 0.02 μM), and incubated for 2 h at 25°C and separated by agarose gel electrophoresis. Lanes 1 ~ 15: pTR80/*Sca*I, lanes 16 ~ 30: pNTR/*Sca*I.

In order to assess the cleavage property of each chimeric endonuclease *in vitro*, the purified protein was added to a reaction mixture including the linearized plasmid DNA containing human (TTAGGG)_80 _telomeric repeats (pTR80) or nontelomeric sequence (pNTR; see Methods). When a chimeric endonuclease has the ability to cleave human telomeric repeats, pTR80 (3.5 kbp), which has (TTAGGG)_80 _repeats in the centre, is expected to be digested into approximately 1.8 kb and 1.2 kb fragments (Figure 2a). We found that our chimeric endonucleases (T-EN, M-EN, EN-T, EN-NM, EN-M, T-FN and FN-T) cut the pTR80 plasmid and produced additional 1.8 kb and 1.2 kb bands (Figure 2b, lanes 4-8, 13 and 14, respectively), although the nonfused proteins (T, M and EN; lanes 2, 3 and 9, respectively), a mutant TRAS1EN protein (ENmut; lane 12) and its chimeric proteins with TRF1 (T-ENmut, ENmut-T; lanes 10, 11) did not show the additional bands for pTR80. Although it has been reported that TRAS1EN cleaves vertebrate telomeric repeats [11], TRAS1EN itself (EN, lane 9) did not cleave pTR80 effectively in this study because of a low concentration of purified TRAS1EN. None of the fused and nonfused proteins showed any extra bands for the nontelomeric plasmid pNTR (Figure 2b, lanes 16-30). Thus, these chimeric EN endonucleases and chimeric FN endonucleases fused with the human telomere binding protein TRF1 or with its Myb domain, specifically and effectively cleaved the human (TTAGGG)_n _telomeric repeats. As far as we know, this is the first report of endonucleases which cleave (TTAGGG)_n _more effectively than other sequences.

### Activity and specificity of the chimeric endonucleases

**Figure 3:**
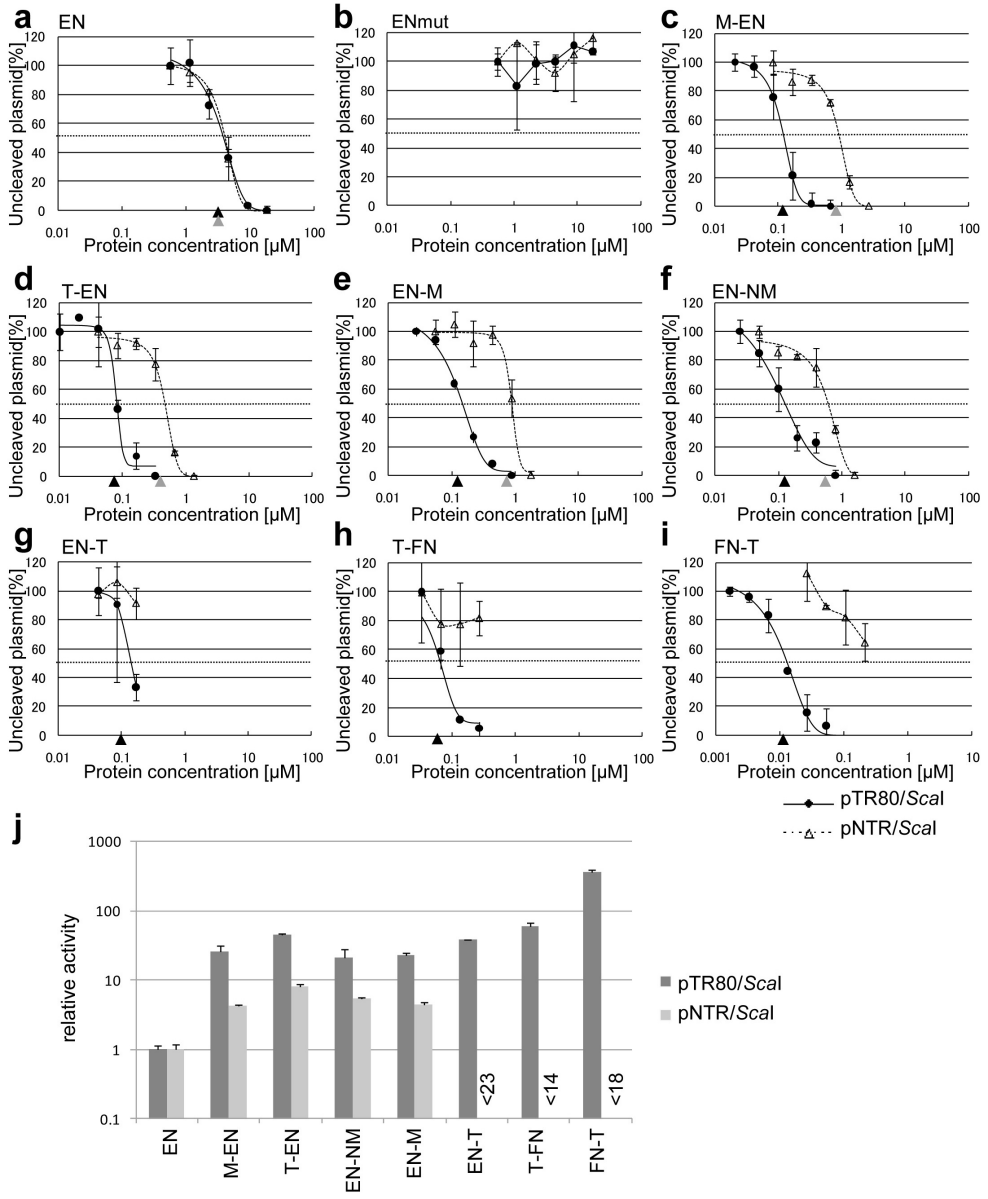
**Comparison of cleavage activity of chimeric endonucleases**. (a-i) pTR80/*Sca*I or pNTR/*Sca*I was mixed with purified protein. Cleaved plasmid was separated by electrophoresis and the intensity of bands corresponding to uncleaved plasmid was determined using a densitometer and ImageJ (bar: ± standard deviation). The cleavage for pTR80 and pNTR is shown by solid line (with filled circles) and dotted line (with open triangles), respectively, in each figure. Protein concentration (μM) is shown on the horizontal axis. EC50 is shown as black arrow head for pTR80 and gray arrow head for pNTR. (j) The reciprocal EC50s of chimeric endonucleases for pTR80/*Sca*I and pNTR/*Sca*I were calculated (bar: ± 95% confidence interval), and each relative activity is shown based on the value of reciprocal EC50 of EN for pTR80/*Sca*I as 1.

We further evaluated the cleavage activity of each chimeric endonuclease as described below. Linearized pTR80 or pNTR was added to a dilution series of protein and incubated at 25°C for 30 min. Then the ratio of cleaved bands to uncleaved linear plasmid was calculated using agarose gel electrophoresis (Figure 3a-i). The EC50, defined as the concentration of protein that cuts half of the plasmid, for pTR80 of nonfused TRAS1EN (EN) was 3.9 μM (Figure 3a). In contrast, the EC50s for pTR80 of chimeric EN endonucleases (Figure 3c, M-EN; Figure 3d, T-EN; Figure 3e, EN-M; Figure 3f, EN-NM; Figure 3g, EN-T) were much lower (0.09-0.19 μM) (Figure 3, Additional file 3), suggesting that purified TRAS1EN has only 2%-5% of the specific cleavage activity of the purified chimeric endonucleases. In order to compare the cleavage activity of each chimeric endonuclease, the relative activities for pTR80 and pNTR were calculated from reciprocals of each EC50 (Additional file 3). The relative activities of five chimeric EN endonucleases (M-EN, T-EN, EN-NM, EN-M and EN-T) for pTR80 were 21-45-fold higher than that of TRAS1EN alone (Figure 3j, Additional file 3). The EC50s for pNTR of EN-T, T-FN and FN-T could not be determined, because the purified protein concentration was too low to induce nonspecific cleavage. FN-T showed the highest *in vitro *cleavage activity for pTR80 because it was 364 times higher than that of TRAS1EN and it cleaved 20 times more telomeric repeats than nontelomeric sequences. EC50s for pTR80 and pNTR of TRAS1EN were the same (Figure 3a). This may appear to be conflicting with our previous report of TRAS1EN being the telomere cleavage endonuclease [11]. This could be explained by the different substrates used in the two studies. While in this study we used the pTR80 plasmid with telomeric repeats accounting for 14% of the substrate DNA, in the previous report (TTAGGG)_n_-oligonucleotides served as substrates requiring a lower enzymatic specificity [11]. Moreover, pTR80 contains human telomeric repeats, whereas the cleavage activity of TRAS1EN is maximum when silkworm telomeric repeat (TTAGG)_n _is the target sequence.

### Binding and cleavage of telomeric repeats by chimeric endonucleases

In order to understand how our chimeric endonucleases cut the telomeric repeats, we analysed cleavage sites in end-labelled oligo DNA TR5 containing (TTAGGG)_5 _repeats, digested with chimeric endonucleases (Additional file 4). We found that chimeric TRAS1EN endonucleases cleaved the T-A junction of the TTAGGG strand and the C-T junction of the CCCTAA strand, which is consistent with the cleavage sites for TRAS1EN [11]. This demonstrates that the specificity of these chimeric endonucleases was not changed even though TRAS1EN was fused with TRF1 domains. Interestingly, the cleavage sites of the two chimeric FN endonucleases (cleavage site (C|CCTAA) in T-FN and (CCCTA|A) in FN-T) on the CCCTAA strand were different from each other. The linker lengths of T-FN and FN-T are quite different, which might affect their respective cleavage sites on the CCCTAA strand [20].

Next, we performed electrophoretic mobility shift assays (EMSAs) to test the binding capacities of chimeric endonucleases to the telomeric repeats. Like TRF1 (T) and its Myb domain (M) [16], all EN and FN chimeric endonucleases bound to the telomeric oligonucleotide TR5 (Additional file 5a), but not to the nontelomeric sequence (NTR) (Additional file 5b), suggesting that chimeric endonucleases recognize telomeric repeats based on the binding specificity of TRF1.

### TRAS1EN fused with TRF1 cuts telomeric repeats in human cells effectively

**Figure 4:**
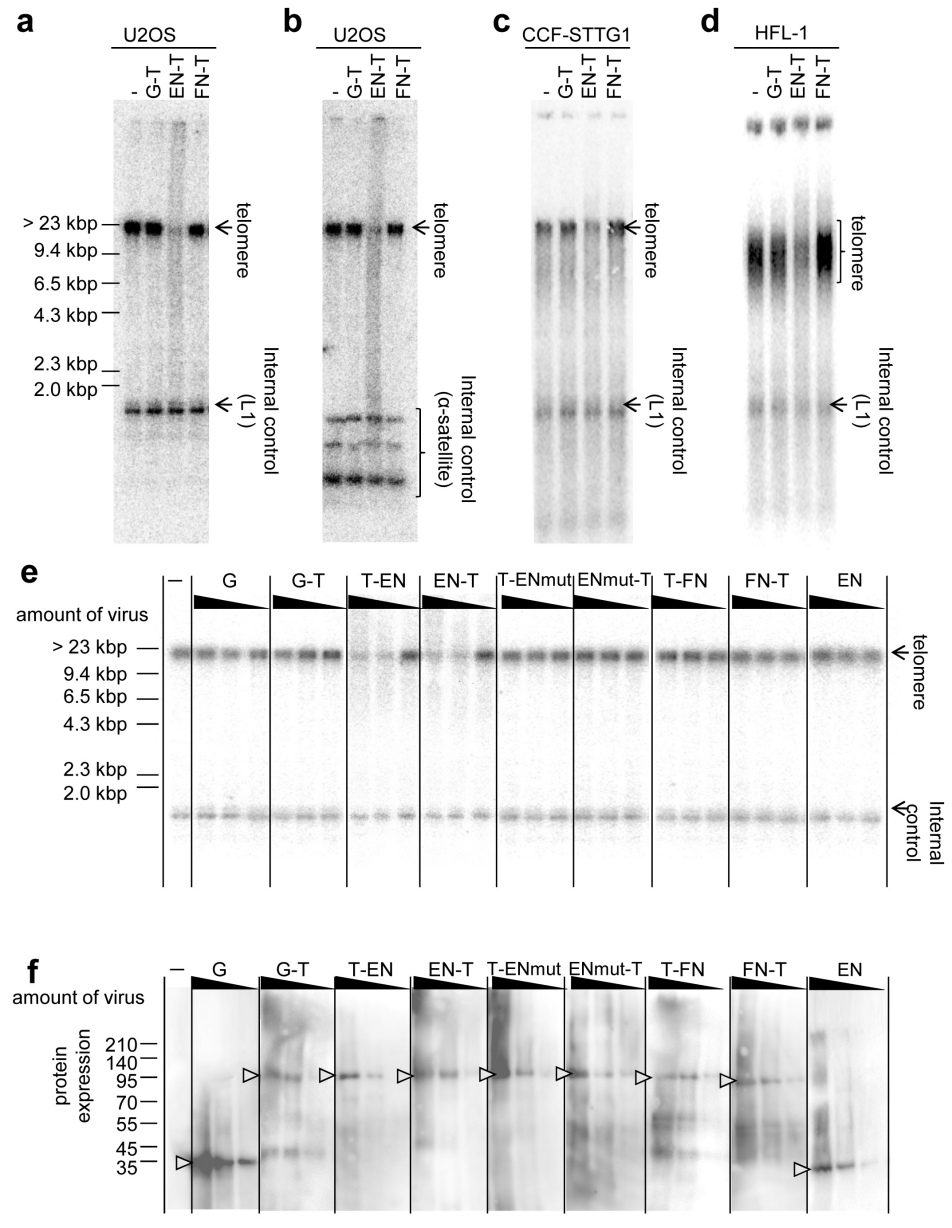
***In vivo *cleavage of human telomere by chimeric endonucleases**. We expressed chimeric endonucleases (G-T, EN-T, FN-T) mediated by adenovirus (MOI: 100) and collected genomic DNA 3 days after infection (see Methods). G-T, GFP-TRF1; -, no virus infection. Using telomeric repeats as a probe, we performed Southern hybridization for terminal restriction fragment (TRF) assay in order to analyse telomere length. We used L1 probe (the sequence is shown in Additional file 6) (a, c and d), and alpha satellite probe (b) as an internal control. (e) Telomere digestion by chimeric endonucleases in U2OS cells. We added each virus construct at MOI 100, 10, 1 (left to right lanes) to the cells, and performed a TRF assay with the same procedure as in (a). G, GFP; other abbreviations (see Figure 1a or text). (f) Protein expression in U2OS cells after adenoviral infections. Expressed proteins used in (e) were confirmed by western blotting using anti-HA antibody. The amounts of expressed proteins are directly proportional to viral titers.

In order to test whether these endonucleases also cleave the telomeric repeats *in vivo*, we introduced endonuclease constructs into two telomerase-negative cancer cell lines, U2OS and CCF-STTG1, and a normal fibroblast cell line HFL-1 using adenovirus vector. Genomic DNA was collected 3 days after virus infection and the telomere length was measured by Southern hybridization (see Methods). In the case of U2OS, CCF-STTG1 and HFL-1 cells, which have long telomeric repeats (over 10 kb), the band intensity of telomeric repeats decreased drastically when EN-T adenovirus was infected, but did not change when GFP-TRF1 (G-T) and FN-T viruses were infected, in comparison with no virus infection (Figure 4a-d). The human L1 sequence and centromeric alpha satellite were used as a nontelomeric internal control for equal loading, and for the comparison of non-specific cleavage, and its banding patterns were not changed in any treatment (Figure 4a-d). These data demonstrate that EN-T was able to cut telomeric repeats specifically and effectively not only *in vitro *but also in human cell lines *in vivo*. Unexpectedly, FN-T, which showed the highest activity *in vitro *(Figure 3j), did not cut the telomeric repeats in human cells. Using U2OS cells showing the most evident telomere digestion in Figure 4a-d, we tested the telomere cleavage by various adenovirus constructs with MOI 100, 10 and 1 (Figure 4e). Two constructs, T-EN and EN-T cleaved telomeric repeats clearly but other constructs, including the TRAS1EN mutant fusion proteins (T-ENmut and ENmut-T), did not alter band intensity. We confirmed that each protein was normally expressed and increased proportionally to the virus titre (Figure 4f).

### Subcellular localization of telomere specific chimeric endonucleases

**Figure 5:**
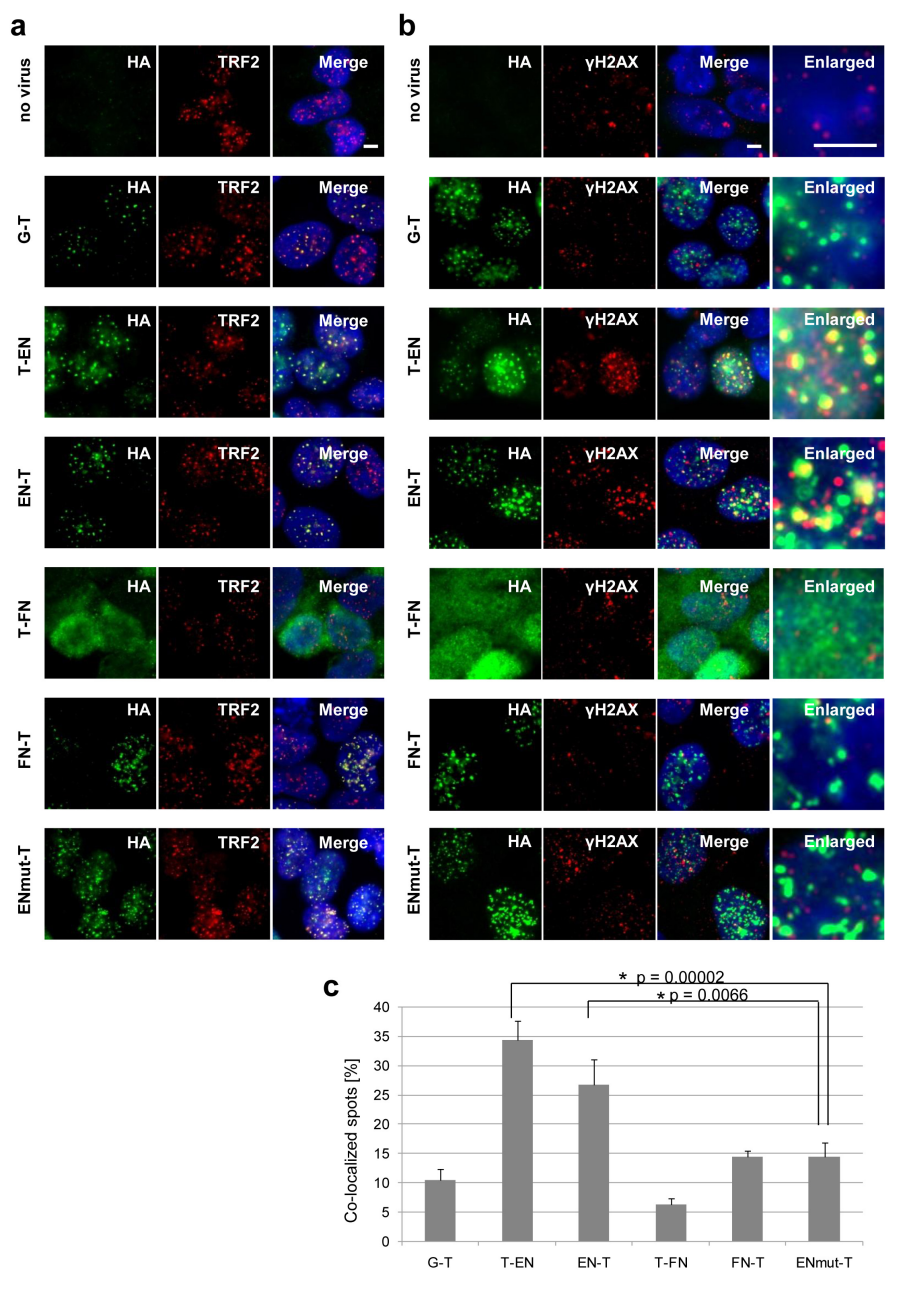
**Subcellular localization of expressed chimeric endonucleases**. (a) Telomere localization of expressed chimeric endonucleases. We infected adenovirus that expresses a chimeric endonuclease at MOI 100 to U2OS cells and performed indirect immunofluorescence the next day. Fusion proteins were detected with anti-HA antibody (green), and telomere was detected with anti-TRF2 antibody (red). Nuclei were stained with Hoechst33342. Yellow dot signals represent the colocalization of fusion protein and telomere. The scale bar is 10 μm. (b) Telomere cleavage by expressed chimeric endonucleases. In order to visualize telomere cleavage by chimeric endonucleases, fusion proteins were detected with anti-HA antibody (green) and DNA double-strand breaks were detected with anti-γ-H2AX antibody (red). (c) Based on the results in (b), we calculated the ratio (%) of co-localized spots (yellow) to fusion protein signals (green) using ImageJ (bar: 95% confidence interval). * shows a statistically significant increase of colocalization to ENmut-T (*P *< 0.01).

As the TRF1 protein is known to accumulate around telomeres [21], we analysed in order to see if our chimeric endonucleases also localize at telomeres. The subcellular localizations of chimeric endonucleases expressed with HA tags were detected with an anti-HA antibody and telomeres were detected with an antibody against TRF2, another component of the shelterin complex [16]. We found that all chimeric endonucleases, except for T-FN, localized at telomeres, as shown by yellow signals in the merged image seen in Figure 5a. The T-FN chimeric endonuclease seemed not to migrate into the nucleus (Figure 5a), which may explain why T-FN could not cut telomeric repeats in cells (Figure 4e).

Indirect immunofluorescence of γH2AX, which is a double-strand-break marker [22], visualized telomere cleavage by chimeric endonucleases in U2OS cells (Figure 5b). Only after infection with the T-EN or EN-T adenovirus did we observe many co-localized spots of chimeric endonuclease and γH2AX (yellow signals in the merged and enlarged images). There were some background γH2AX signals in PML bodies [22,23] without virus infection. Using NIH Image J software, we quantified colocalization of HA and γH2AX signals (Figure 5c) and found that 34% of T-EN and 27% of EN-T foci localized at double-strand breaks. These observations confirmed that both T-EN and EN-T localize at telomeres and cut telomeric DNA.

### Inhibition of cell proliferation

**Figure 6:**
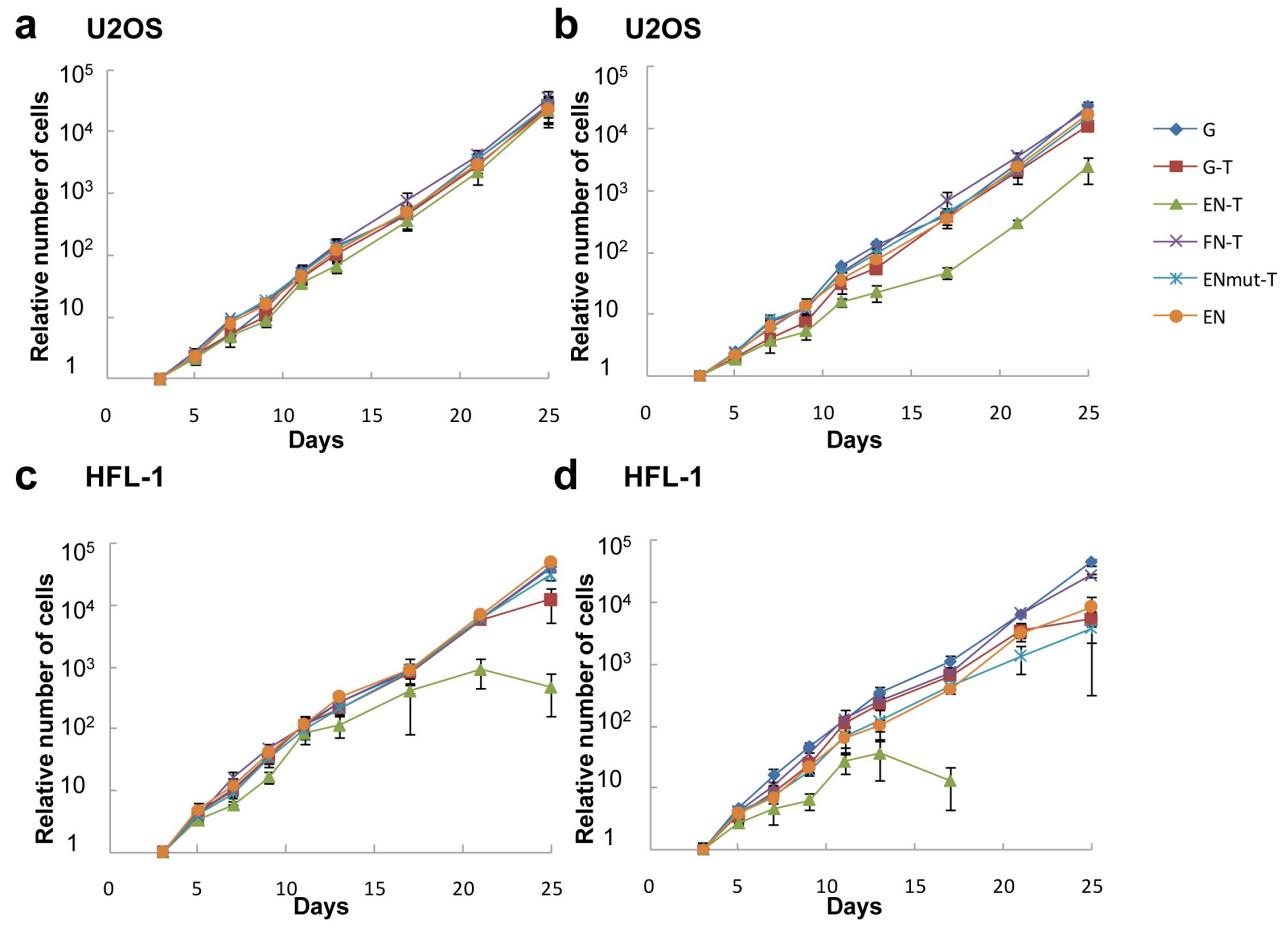
**Inhibition of cell proliferation by an expressed EN-T endonuclease**. A series of U2OS and HFL-1 cells were planted in 12 well plates (3 × 10^5 ^per well). We added recombinant adenovirus to 1 ml medium at low concentrations of 4.4 × 10^5 ^pfu/ml (a), (c) or 8.8 × 10^5 ^pfu/ml (b), (d). Cell growth curves for U2OS (a), (b) and HFL-1 (c), (d) are shown (bar: ± standard deviation, *n *= 4). Numbers of cells at 3 days after infection are indicated as 1.0. EN-T expressing HFL-1 cell cultures died by day 17.

We examined whether the telomere shortening induced by chimeric EN endonucleases could repress the cell proliferation directly. A low concentration of recombinant adenoviruses was added to U2OS and HFL-1 human cells at each passage, every 3-4 days. Twenty-five days after viral infection, the growth of U2OS tumour cells infected with EN-T-expressing adenovirus was inhibited at the higher dose (Figure 6b). In contrast, U2OS cells infected with adenoviruses expressing chimeric proteins, G-T, FN-T, ENmut-T and EN showed normal growth similar to the cells expressing only GFP (G). We further analysed HFL-1 fibroblasts, which have no telomerase activity to elongate shortened telomere. The growth of HFL-1 cells infected with EN-T virus was more clearly inhibited than that of U2OS with the same virus (Figure 6c, d). HFL-1 cell cultures infected with EN-T adenovirus titres of 8.8 pfu/ml were dead by day 17 after infection.

**Figure 7:**
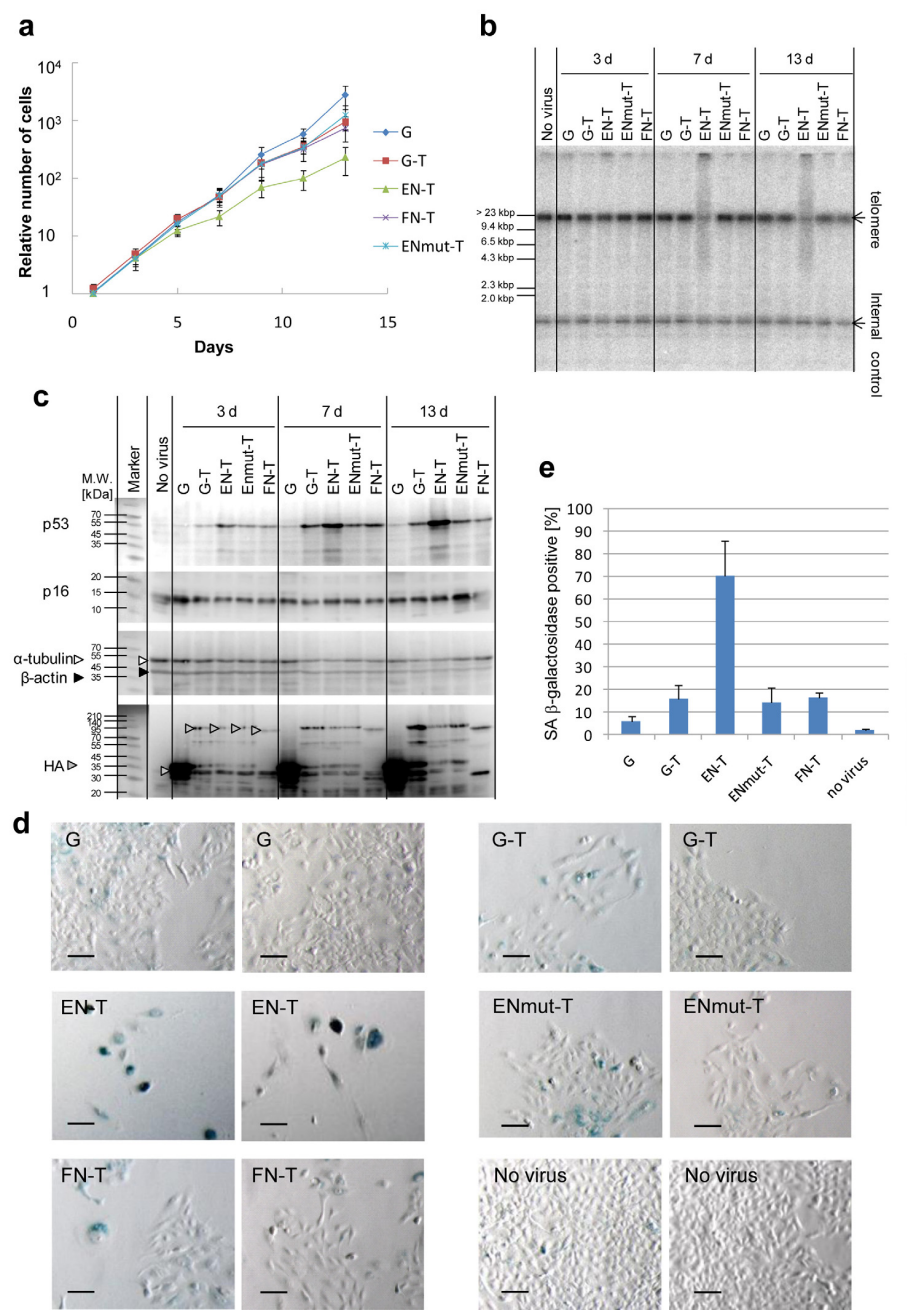
**Analysis of the inhibition of U2OS cancer cell growth**. We added recombinant adenovirus at the concentration of 8.8 × 10^5 ^pfu/ml to the medium. (a) Numbers of U2OS cells after infection of each virus construct. Cell numbers at every 2 days starting from 1 day after infection are shown. Cell number at 1 day after infection is indicated as 1.0. (b) Telomere digestion by expressed chimeric endonucleases. We performed TRF assay with U2OS cells 3, 7 and 13 days after viral infection. (**c**) Immunoblot analysis of U2OS cell extracts harvested 3, 7 and 13 days after viral infections. p53 and p16 are markers of cellular senescence. Anti-HA antibody (HA) was used to detect chimeric endonucleases. α-tubulin and β-actin expression were used as loading controls. (d, e) We performed a senescence-associated (SA) β-galactosidase assay (see Methods) on day 13 cells. EN-T expressing cells show senescent phenotype, that is, an enlarged and flattened appearance and increased SA β-galactosidase activity as shown in (d) (two different fields are shown for each sample; the scale bar is 100 μm), and the percentage of the SA β-galactosidase positive cells is shown in (e) (bar: 95% confidence interval).

Then we explored the senescence status of U2OS tumour cells expressing chimeric EN endonuclease. Consistent with the results presented in Figure 6b, EN-T expression suppressed proliferation of U2OS cells 2 weeks after infection, while ENmut-T did not exhibit such suppression effect (Figure 7a). We measured telomere length at day 3, 7 and 13 after infection to analyse telomere digestion by EN-T and other expression products (Figure 7b). Telomere digestion was detected from day 7 with the EN-T virus, while not with other viruses including ENmut-T. We also compared the gene expression levels of p53 and p16INK4a (p16) after viral infection (Figure 7c). The p53 and p16 proteins are known as key factors in the telomere DNA damage response. In the experiment of telomere loop structure disruption by overexpression of dominant negative TRF2 (TRF2^ΔBΔM^), p53 was induced within a few days and p16 was induced after 8-10 days [24]. The p53 and p16 proteins induced cellular senescence independently by inhibiting their downstream target cyclin-dependent kinase [25]. In this experiment, the expression of p53 was induced with EN-T virus 7 days after viral infection, but the level of p16 remained stable (Figure 7c). ENmut-T expression also activated p53 slightly, this could be because TRF1-overexpression affected cell cycle progression [26]. Nevertheless, EN-T expression clearly has the strongest effect on p53 activation. It was confirmed that the amount of EN-T protein was almost equal to that of ENmut-T (Figure 7c, HA). We also performed a senescence-associated (SA) β-galactosidase assay in order to detect cellular senescence. EN-T-expressing cells showed senescent phenotype, an enlarged and flattened appearance and increased SA β-galactosidase activity (Figure 7d). We found that 70% of cells infected with EN-T virus were positive for SA β-galactosidase staining, while only 14% with the ENmut-T virus (Figure 7e). These results indicate that EN-T virus induces cellular senescence through telomere shortening and represses cell proliferation.

## Discussion

Type IIS restriction endonucleases (REases) such as *Fok*I and *Bmr*I, which have a specific DNA binding domain and a nonspecific DNA cleavage domain separately, are useful to create new sequence-specific endonucleases [18,27]. For this purpose, DNA binding domains such as the Z-conformation-specific Za domain of human RNA adenosine deaminase, Gal4 and zinc finger motifs are fused with the *Fok*I DNA cleavage domain (FN). The most extensively studied group of chimeric nucleases is based on zinc finger nucleases (ZFNs), because zinc finger motifs can be designed to bind a large range of DNA sites [28]. It was reported that ZFNs could cleave their chromosomal targets in frog oocytes, *Drosophila*, plant cells, *Caenorhabditis elegans *and human cells [29,30]. However, so far, there has been no report of telomere specific endonuclease being created. The cleavage domain of type IIS REase has no specificity for any target sequence, but the endonuclease domains found in several target-specific LINEs are highly sequence-specific. Thus, the coupling of such a LINE endonuclease with a DNA-binding protein will produce an endonuclease with improved site-specificity.

To cut human (TTAGGG)_n _telomeric repeats specifically, we first tried to use TRAS1EN (EN), the endonuclease domain from the silkworm telomere-specific LINE TRAS1, but it had insufficient activity and specificity. Therefore, we tried to make chimeric endonucleases of EN or FN fused with the human telomeric repeat binding protein TRF1. Five chimeric EN endonucleases and two chimeric FN endonucleases cut the telomeric repeats (pTR80) specifically but did not cut the nontelomeric sequence in the same enzyme concentration (pNTR; Figures 2, 3). There was no major difference in specificity for pTR80 with various constructs (Figure 3) which suggested that the domain constituents and structures of chimeric endonucleases do not affect their activities, and that the TRF1 Myb domain included in all chimeric enzymes is essential for cleavage specificity to human telomeric repeats. FN-T and T-FN digested both strands of (TTAGGG/CCCTAA)_n _repeats with different cleavage sites on the top strand, but chimeric enzymes based on TRAS1EN digested both strands specifically as does TRAS1EN itself (Additional file 4), so the specificity of TRAS1EN is left unaffected even in the context of chimeric constructs. The EC50 for cutting telomeric repeat DNA of FN-T, the most active enzyme *in vitro*, was 0.011 μM and the value of EN-T was 0.10 μM (Figure 3j). Both values seem low enough to be functional in the cell, in comparison with other active enzymes such as ZFN [18] (EC50, ~0.01 μM) and artificial homing endonuclease [14] (EC50, ~0.2 μM).

When these chimeric endonucleases were expressed in cancer cells, T-EN and EN-T moved to telomeres (Figure 5a) where they induced double-strand breaks (Figure 5b, c) and telomere digestion (Figure 4e). In contrast, T-FN could not move into the nucleus (Figure 5a) and T-FN and FN-T showed insufficient double-strand breaks at telomere (Figure 5c). Thus, neither produced telomere digestion *in vivo *(Figure 4a, c), although they have strong activities *in vitro*. T-FN may lose its NLS function within the TRF1 region because of conformational changes. In addition, there are several reports of chromosomal double strand breaks *in vivo *by FN chimeric endonuclease [29,30] but the prokaryotic enzyme FN might have less endonuclease activity for telomeres with a eukaryotic chromatin structure than the telomere-specific endonuclease TRAS1EN.

Through telomere shortening, the cells expressing EN-T, showed higher levels of cellular senescence markers (Figure 7c-e) and suppressed cell proliferation (Figure 7a). Thus, this chimeric endonuclease has the potential to be used as anticancer reagents. During the progression of cancer, one of the important mechanisms is telomerase activation which circumvents the telomere-dependent pathways of replicative senescence and crisis [4]. The lack of telomerase expression in most normal somatic cells makes this enzyme an attractive target when designing anticancer therapeutics. Thus, the inhibition of telomerase by a dominant negative hTERT mutant in human acute leukemia cells caused not only telomere shortening but also induction of apoptosis [31]. However, it took more than 40 days and such an approach would be valid only in the telomerase positive cells. Here, we have succeeded in developing a novel approach to shorten telomeres directly and quickly by TRAS1EN chimeric endonucleases. Compared with telomerase inhibitors, which are not effective in telomerase-negative cancer cells [30], these TRAS1EN chimeric enzymes have the great advantage of being effective not only in the telomerase-positive cancer cells but also in telomerase-negative cancer cells such as U2OS. Future modifications of the expression vectors using cancer-specific enhancer-promoters, such as the hTERT promoter and the hypoxia-inducible enhancer [32], to restrict the expression of the chimeric endonuclease in specific cancer cells could reduce unwanted effects on normal cells. This paper shows the concept of using such chimeric endonuclease to inhibit the growth of cancer cells by telomeric repeat shortening.

## Conclusions

We created novel chimeric endonucleases to cut human telomeres by fusing the endonuclease domain of the silkworm's telomere specific non-LTR retrotransposon TRAS1 to the human telomere-binding protein, TRF1. These chimeric endonucleases were able to cut telomeric repeats specifically and effectively not only *in vitro *but also in human cell lines *in vivo*, and EN-T-expressing adenovirus suppressed the cell growth of cancer cells in a few weeks. This report shows the possibility of cancer gene therapy by rapid telomere shortening.

## Methods

### Cloning of TRF1, TRAS1EN and FN in the expression vector

Three-dimensional modelling of chimeric proteins was performed with PyMol v0.99-rc6 http://www.pymol.org. We started from the co-crystal structure of the Myb domain of TRF1 and DNA (PDB ID: 1W0T), then overlaid EN (PDB ID: 1WDU) or FN (PDB ID: 1FOK). Although the co-crystal structure of EN and DNA has not yet been clarified, the interaction between EN and DNA was surmized via homology modelling using the crystal structure of DNaseI (PDB ID: 1DNK) [11]. The TRF1, TRF1 Myb domain, and TRF1 NLS-Myb domain were amplified by polymerase chain reaction (PCR) with Pfu Turbo DNA polymerase (Stratagene, CA, USA) using primers TRF1-f-Nco-Nhe, TRF1M-f-Nco-Nhe, TRF1NM-f-Nco-Nhe and TRF1-b-Sal-Not (Additional file 6) from HEK293 cell cDNA. EN and EN(H258A) were amplified using primers T1EN-f-Sal-Nhe and T1EN-b-Nco-Not (Additional file 6) from plasmid pHisT1EN or pHisT1EN(H258A) [9] and FN was amplified using primers FN-f-Sal-Nhe and FN-b-Nco-Not (Additional file 6) from plasmid pML109RM119-1 (ATCC biological resource centre). All primers were designed to contain restriction endonuclease sites and PCR products were digested with the corresponding enzymes (Figure 1). The products were cloned into the pET21b expression vector (Novagen), which is an expression vector for *E. coli *and contains a His tag in the *C*-terminal end. To produce chimeric constructs, in the case of pET21b-EN-T vector, TRF1 was subcloned into pET21b digested with *Nhe*I and *Not*I, and then TRAS1EN was subcloned into pET21b-TRF1 digested with *Nhe*I and *Nco*I. Other vectors were constructed similarly. The insert sequence of a candidate clone was confirmed. Adenovirus expression vectors were constructed from pAxCAwtit (TAKARA), which contains the CAG (chicken beta-actin promoter and cytomegalovirus immediate-early enhancer) promoter. Insert sequences were amplified from each pET21b vector using 5'-phosphorylated primers Kozak-HA-rbs and His tag (Additional file 6) and were ligated into pAxCAwtit digested with *Swa*I. Recombinant adenoviruses were generated according to the manufacturer's procedures. Recombinant pAxCAwtit vectors were digested with *Nsp*V and the linearized adenovirus DNAs were transfected into human 293 cells. Recombinant adenoviruses were generated in 293 cells. The map of adenovirus vector is shown in Additional file 7.

### Expression and purification of chimeric endonucleases

The plasmids containing chimeric endonucleases were transformed into the *E. coli *strain Rosetta2(DE3)/pLysS (Novagen) plus pG-KJE8 (TAKARA). We used the Rosetta2 *E. coli *strain because this strain carried a plasmid containing tRNA genes to decode rare *E. coli *codons, and the strain was designed to enhance the expression of eukaryotic proteins. The plasmid pG-KJE8, which contains chaperons to facilitate protein folding and enhance production of active proteins, was co-transformed. Transformants were grown in 10 ml LB containing 50 μg/ml ampicillin, 20 μg/ml chloramphenicol, 100 μg/ml kanamycin, 5 ng/ml tetracycline, and 0.5 mg/ml L-arabinose culture at 37°C until the optical density at 600 nm (OD600) reached 0.6. Isopropyl-β-D-thiogalactopyranoside (IPTG) was then added to a final concentration of 1 mM, and incubation was continued for 12 h at 25°C. Protein purification was carried out according to the manufacturer's protocol for Ni-NTA agarose (Qiagen, CA, USA). Glycerol up to a concentration of 5% was added to the eluted proteins which were stored at -80°C. Eluates were run on SDS-PAGE and the target proteins were detected as bands of predicted molecular masses. We calculated the percentage of the target protein in the total eluted proteins from Additional file 2b by quantification with ImageJ v. 1.34s (National Institutes of Health [NIH], MD, USA). The concentrations of the total eluted proteins were determined by Bradford assay using Proteostain Kit (Dojindo, MD, USA).

### Assays for endonucleolytic activities

The telomeric repeat plasmid pTR80 was constructed from a PCR product of an extended primer dimer of (TTAGGG)_5 _and (CCCTAA)_5 _primers. A non-telomeric repeats (NTR) sequence was randomly selected from human mRNA, and 2022-2672 bp of Tat-SF1 (GenBank accession number U76992) was amplified with primers dT18 and NTR-f (Additional file 6) and cloned into the pGEM T-Easy vector (Promega, WI, USA). For quantitative chimeric endonuclease activity assays, plasmid pTR80 and pNTR were digested with the restriction endonuclease *Sca*I to make a linear substrate with telomeric repeats positioned in the centre of the DNA strand. Then, DNA was purified using the PCR clean-up kit (Sigma, NY, USA). DNA concentration was determined spectrophotometrically at 260 nm. Chimeric endonuclease was added to 10 μl of reaction buffer containing 1.6 ng/μl substrate DNA. Samples were incubated for 1 h at 25°C, and the reaction products were analysed by agarose gel electrophoresis. Ethidium bromide-stained gels were digitized with PrintGraph (Atto, Tokyo, Japan). The intensity of the product bands was quantitated using ImageJ v. 1.34s (NIH). Assays for oligonucleotide endonucleolytic activities were performed as described previously [9]. Oligonucleotides were labelled at the 5' end by the enzymatically catalyzed transfer of ^32^P from [γ-^32^P] adenosine triphosphate (ATP) with T4 polynucleotide kinase. The oligonucleotide concentration was fixed at 100 nM with adding nonlabelled oligonucleotides. The reaction buffer for chimeric endonucleases contained 5 mM HEPES-KOH (pH 7.9), 10 mM NaCl, 2 mM MgCl_2_, 100 μg/ml BSA and 500 nM non-specific oligonucleotide, NTR to suppress non-specific binding of misfolded chimeric endonuclease to non-telomeric sequence. This mixture was treated with a purified chimeric endonuclease in 10 μl of reaction buffer for 1 h at 25°C. The reaction mixture was denatured in a loading buffer containing 75% formamide for 5 min at 95°C, immediately chilled on ice and run on a 28% Long Ranger polyacrylamide denaturing sequencing gel (Lonza, Basel, Switzerland). Quantitation of the reaction products was carried out with a BAS 5000 imaging analyser system (Fujifilm).

Various-sized telomeric repeat oligonucleotides were end-labelled with T4 polynucleotide kinase and [γ-^32^P] ATP and were used as molecular size markers: dG8, dG14, dG20, dG26, dC9, dC15, dC21 and dC27 (Additional file 6).

### Electrophoretic mobility-shift assay

DNA probes were 5'-end-labelled as described earlier [9], and were gel-purified. About 15 fmol of DNA was labelled in a 10 μl reaction and the final probe concentration in binding assays was <10 nM. Binding reactions were carried out by incubating the indicated amounts of protein (see figure legends for details) in 10 μl of a reaction mix of 20 mM Hepes (pH 7.9), 150 mM KCl, 0.1 mM EDTA, 1 mM DTT, 4% Ficoll, 5% glycerol, 0.1 mg/ml of BSA and 3 ng/μl salmon sperm DNA. Samples were incubated at 4°C for 60 min and then run on native 5% polyacrylamide gels as described [33].

### Cell cultures and adenovirus production

Two hundred and ninety-three U2OS, CCF-STTG1 and HFL-1 cells were cultured in Dulbecco's modified Eagle's medium with 10% fetal bovine serum [34]. All cell lines were provided from the RIKEN BioResource Center (Ibaraki, Japan). Cells were routinely passaged at 80% confluence. Adenoviruses were used as freeze-thaw lysates, and their titres were estimated following the manufacturer's procedure of the Adenovirus Expression Vector Kit (TAKARA, WI, USA) for each virus (*n *= 16). Transfections were performed with FuGene HD (Roche) according to the manufacturer's procedure. Four separate microscope images were used for counting the number of cells with CellProfiler [35].

### Telomere length measurement

Genomic DNA was purified with PUREGENE (Gentra, WA, USA) and digested with HinfI. Digests were separated on a native 0.8% agarose gel, blotted in 0.4 M NaOH onto a Hybond N+ membrane (Amersham, Uppsala, Sweden) and hybridized with the RI-labelled (CCCTAA)_5 _and 1490-1467 bp of L1 (Additional file 6; GenBank accession number X52235.1) probes in hybridization buffer (6× saline-sodium citrate [SSC], 1% sodium dodecyl sulphate [SDS], 5× Denhardt's solution, 2.5 μg/ml salmon sperm DNA) at 37°C for 12 h. The membrane was washed several times with wash buffer (4× SSC, 0.1% SDS) for 10 min at 37°C. Quantitation of the reaction products was carried out with a BAS 5000 imaging analyser system (Fujifilm).

### Immunoblotting analysis

Cells were trypsinized, washed once with PBS and subsequently lysed in 200 mM Tris-HCl pH 6.8, 4% SDS, 20% glycerol, 200 μM DTT and 0.2% bromophenol blue. Lysates were separated on SDS polyacrylamide gels (29:1 acrylamide: bisacrylamide, 8% gel) and transferred onto BioTrace PVDF (Pall) for 70 min at 14 V. Membranes were preincubated in TBST (0.1% Tween20 in 1× TBS) containing 5% BSA for 30 min and then incubated with primary antibodies in Can Get Signal Solution 1 (TOYOBO, Osaka, Japan) for 1 h, followed by three 10 min washes with TBST. The following antibodies were used: TRF2, IMGENEX #IMG-124A; γ-H2AX, LPBIO #AR-0149; HA, BETHYL #A190-108A; p53, EXBIO #11-114-C100; p16INK4a, Thermo #MS-383-P0; α-Tubulin, CEDARLANE #CLT9002 and β-Actin, Abcam #ab8226. Membranes were incubated for 1 h with horseradish peroxidase conjugated anti-mouse or anti-rabbit antibodies (Amersham) in Can Get Signal Solution 2 (TOYOBO) and were developed using the ECL PLUS system (Amersham). All samples were loaded in the same gel and the blotted membrane was re-stained with different antibody after stripping in 2% SDS, 100 mM β-mercaptoethanol and 62.5 mM Tris-HCl pH 7 at 50°C for 30 min.

### Immunofluorescence

The cellular localization of chimeric endonucleases was visualized through indirect immunofluorescence. Cells were grown on glass-bottomed dishes, washed once with PBS, fixed with methanol, and permeabilized in a Triton X-100 solution (0.5% Triton X-100 in phosphate-buffered saline) four times for 10 min. The cells were subsequently blocked for 1 h at 25°C in 10% sheep serum, stained with primary or fluorescence-conjugated secondary antibodies for 1 h at 37°C, and then visualized under an Olympus IX51 fluorescence microscope. Anti-TRF2 antibody (IMGENEX, CA, USA; #IMG-124A) was used to detect telomere, because TRF2 is another telomere binding protein being independent of TRF1, and anti-HA antibody (BETHYL, TX, USA; #A190-108A) was used to detect chimeric endonucleases. Secondary antibodies were Alexa Fluor 488 or 555-conjugated goat anti-mouse or anti-rabbit antibodies (Molecular Probes, CA, USA).

### Senescence-associated beta-galactosidase staining

Senescence-associated beta-galactosidase (SA β-gal) activity at pH 6.0 is a commonly used cellular senescence marker [36]. Positive cells were detected by the modified method of Dimri *et al*. [36]. The monolayers of cells were washed with PBS and then fixed with 1% glutaraldehyde for 5 min. The cells were washed again twice with PBS, then staining solution was added [1 mg/ml 5-bromo-4-chloro-3-inolyl-b-D-galactoside (X-gal), 40 mM sodium phosphate, pH 6.0, 5 mM potassium ferrocyanide, 5 mM potassium ferricyanide, 150 mM NaCl, 2 mM MgCl_2_]. After the cells were further incubated at 37°C for 24 h, the percentage of stained cells was counted. The number of blue structures was counted in four fields (from a total of average 3.76 × 10^3 ^cells).

## Abbreviations

AP: apurine/apyrimidine; APT: adenosine triphosphate; EN: endonuclease-like domain; FN: *Fok*I cleavage domain; LINE: long interspersed nuclear element; LTR: long terminal repeat; NLS: nuclear localization signal; NTR: non-telomeric repeat; PBS: phosphate buffered saline; PCR: polymerase chain reaction; SA: senescence-associated; SDS: sodium dodecyl sulfate; SSC: saline-sodium citrate; ZFN: zinc finger nuclease.

## Competing interests

The authors declare that they have no competing interests.

## Authors' contributions

KY carried out the molecular genetic studies and contributed to the manuscript. HA participated in the design of the study. HF conceived and coordinated the study and helped to write the manuscript. All authors read and approved the final manuscript.

## Supplementary Material

Additional file 1 **Figure S1 - The amino acid sequences of protein linkers**. The protein linker sequences of T-EN, M-EN, EN-T, EN-NM, EN-M, T-FN and FN-T are shown. The second structures of these proteins are predicted by SSpro http://scratch.proteomics.ics.uci.edu/. 67 -267 a.a. of the linker of EN-T and FN-T are TRF Homology domain (TRFH) which has tight structure, so we calculated linker length except this domain. Most of these linkers are nonstructural peptide. Click here for file

Additional file 2 **Figure S2 - SDS-PAGE of purified chimeric endonucleases**. (a) Selection of an appropriate E. coli strain for the effective expression of human protein (TRF1). Recombinant TRF1 protein was purified with a His tag. Protein extracts produced from bacterial cells were purified using Ni-NTA (nitrilotriacetic acid) agarose beads, and separated on a sodium dodecyl sulphate (SDS)-PAGE gel and stained with Coomassie brilliant blue. We confirmed that the molecular mass of overexpressed protein is identical to TRF1 (53.6 kDa). * indicates non-specific protein bound to Ni-NTA beads. The expression of human protein TRF1 increased with chaperon and rare tRNA. (b) SDS-PAGE of purified chimeric endonucleases using Rossetta2/pLysSRARE2 (Kmr) strain in (a). The molecular weight of each purified protein was the same as the calculated weight. Arrows shows purified protein. Click here for file

Additional file 3 **Table S1**. Cleavage activity of chimeric endonucleases. Click here for file

Additional file 4 **Figure S3 - Cleavage of telomeric repeats by chimeric endonucleases**. (a, b) RI-labelled double-strand oligo DNA substrate, TR5 containing 5 TTAGGG repeats was digested with chimeric endonuclease, and separated on polyacrylamide gels. The DNA concentration was 100 nM, and protein concentration was about 0.3 μM except for EN (6 μM), and FN-T (0.02 μM). The (TTAGGG)_5 _strand of TR5 was labelled in (a) and the (CCCTAA)_5 _strand was labelled in (b). Black arrowheads show major cleavage bands which were cleaved in every telomeric repeat; gray arrowheads show minor cleavage bands. (c) Schematic cleavage sites are shown based on the results of (a) and (b). Click here for file

Additional file 5 **Figure S4 - Binding activity of chimeric endonucleases to telomeric repeats**. In order to examine the binding capacity of chimeric endonuclease to the telomeric repeats, we performed electrophoretic mobility shift assays. Approximately 1 nM RI-labelled double-strand oligo DNA was added with fusion protein at 0.04, 0.2, 1 μM except for T-FN (0.02, 0.1, 0.5 μM) and FN-T (0.01, 0.07, 0.3 μM) from left to right in each gel. Target oligo DNA used as probe was TR5, (TTAGGG)_5 _in (a), and NTR in (b). Click here for file

Additional file 6 **Table S2**. Primer list. Click here for file

Additional file 7 **Figure S5 - The map of recombinant adenovirus genome**. The maps of recombinant adenoviruses, Ad-G, Ad-EN, Ad-GT, Ad-T-EN, Ad-EN-T, Ad-ENmut-T, Ad-T-FN and Ad-FN-T are shown. pAxCAwtit vector contains adenovirus genome between NspV sites. Adenovirus vectors are replication defective by deletion of the E1A and E1B genes which are required for virus replication in mammalian cells, and lack E3 gene which is not essential for virus replication in 293 cells. Recombinant proteins have HA epitope tag at the *N*-terminal end. Click here for file

## References

[B1] Blackburn E Telomere states and cell fates Nature 2000 408 6808 53 56 10.1038/35040500 11081503

[B2] Blackburn EH Structure and function of telomeres Nature 1991 350 6319 569 573 10.1038/350569a0 1708110

[B3] Harley CB Futcher AB Greider CW Telomeres shorten during ageing of human fibroblasts Nature 1990 345 6274 458 460 10.1038/345458a0 2342578

[B4] Kim NW Piatyszek MA Prowse KR Harley CB West MD Ho PL Coviello GM Wright WE Weinrich SL Shay JW Specific association of human telomerase activity with immortal cells and cancer Science 1994 266 5193 2011 2015 10.1126/science.7605428 7605428

[B5] Shin-Ya K Novel antitumor and neuroprotective substances discovered by characteristic screenings based on specific molecular targets Biosci Biotechnol Biochem 2005 69 5 867 872 10.1271/bbb.69.867 15914903

[B6] Wang ES Wu K Chin AC Chen-Kiang S Pongracz K Gryaznov S Moore MA Telomerase inhibition with an oligonucleotide telomerase template antagonist: *in vitro *and *in vivo *studies in multiple myeloma and lymphoma Blood 2004 103 1 258 266 10.1182/blood-2003-02-0546 12969977

[B7] Kojima K Fujiwara H Evolution of target specificity in R1 clade non-LTR retrotransposons Mol Biol Evol 2003 20 3 351 361 10.1093/molbev/msg031 12644555

[B8] Okazaki S Ishikawa H Fujiwara H Structural analysis of TRAS1, a novel family of telomeric repeat-associated retrotransposons in the silkworm, Bombyx mori Mol Cell Biol 1995 15 8 4545 4552 7623845 10.1128/mcb.15.8.4545 PMC230694

[B9] Anzai T Takahashi H Fujiwara H Sequence-specific recognition and cleavage of telomeric repeat (TTAGG)(n) by endonuclease of non-long terminal repeat retrotransposon TRAS1 Mol Cell Biol 2001 21 1 100 108 10.1128/MCB.21.1.100-108.2001 11113185 PMC88784

[B10] Takahashi H Fujiwara H Transplantation of target site specificity by swapping the endonuclease domains of two LINEs EMBO J 2002 21 3 408 417 10.1093/emboj/21.3.408 11823433 PMC125841

[B11] Maita N Anzai T Aoyagi H Mizuno H Fujiwara H Crystal structure of the endonuclease domain encoded by the telomere-specific long interspersed nuclear element, TRAS1 J Biol Chem 2004 279 39 41067 41076 10.1074/jbc.M406556200 15247245

[B12] Maita N Aoyagi H Osanai M Shirakawa M Fujiwara H Characterization of the sequence specificity of the R1Bm endonuclease domain by structural and biochemical studies Nucleic Acids Res 2007 35 12 3918 3927 10.1093/nar/gkm397 17537809 PMC1919474

[B13] Feng Q Schumann G Boeke J Retrotransposon R1Bm endonuclease cleaves the target sequence Proc Natl Acad Sci USA 1998 95 5 2083 2088 10.1073/pnas.95.5.2083 9482842 PMC19257

[B14] Ashworth J Havranek JJ Duarte CM Sussman D Monnat RJ Jr Stoddard BL Baker D Computational redesign of endonuclease DNA binding and cleavage specificity Nature 2006 441 7093 656 659 10.1038/nature04818 16738662 PMC2999987

[B15] Repanas K Zingler N Layer L Schumann G Perrakis A Weichenrieder O Determinants for DNA target structure selectivity of the human LINE-1 retrotransposon endonuclease Nucleic Acids Res 2007 35 14 4914 4926 10.1093/nar/gkm516 17626046 PMC1950540

[B16] Broccoli D Smogorzewska A Chong L de Lange T Human telomeres contain two distinct Myb-related proteins, TRF1 and TRF2 Nat Genet 1997 17 2 231 235 10.1038/ng1097-231 9326950

[B17] van Steensel B de Lange T Control of telomere length by the human telomeric protein TRF1 Nature 1997 385 6618 740 743 10.1038/385740a0 9034193

[B18] Kim YG Cha J Chandrasegaran S Hybrid restriction enzymes: zinc finger fusions to FokI cleavage domain Proc Natl Acad Sci USA 1996 93 3 1156 1160 10.1073/pnas.93.3.1156 8577732 PMC40048

[B19] Arai R Ueda H Kitayama A Kamiya N Nagamune T Design of the linkers which effectively separate domains of a bifunctional fusion protein Protein Eng 2001 14 8 529 532 10.1093/protein/14.8.529 11579220

[B20] Li L Chandrasegaran S Alteration of the cleavage distance of FokI restriction endonuclease by insertion mutagenesis Proc Natl Acad Sci USA 1993 90 7 2764 2768 10.1073/pnas.90.7.2764 8464886 PMC46176

[B21] Broccoli D Chong L Oelmann S Fernald A Marziliano N van Steensel B Kipling D Le Beau M de Lange T Comparison of the human and mouse genes encoding the telomeric protein, TRF1: chromosomal localization, expression and conserved protein domains Hum Mol Genet 1997 6 1 69 76 10.1093/hmg/6.1.69 9002672

[B22] Takai H Smogorzewska A de Lange T DNA damage foci at dysfunctional telomeres Curr Biol 2003 13 17 1549 1556 10.1016/S0960-9822(03)00542-6 12956959

[B23] Mirzoeva O Petrini J DNA damage-dependent nuclear dynamics of the Mre11 complex Mol Cell Biol 2001 21 1 281 288 10.1128/MCB.21.1.281-288.2001 11113202 PMC88801

[B24] Jacobs J de Lange T p16INK4a as a second effector of the telomere damage pathway Cell Cycle 2005 4 10 1364 1368 16177573 10.4161/cc.4.10.2104

[B25] Deng Y Chan S Chang S Telomere dysfunction and tumour suppression: the senescence connection Nat Rev Cancer 2008 8 6 450 458 10.1038/nrc2393 18500246 PMC3688269

[B26] Ohki R Ishikawa F Telomere-bound TRF1 and TRF2 stall the replication fork at telomeric repeats Nucleic Acids Res 2004 32 5 1627 1637 10.1093/nar/gkh309 15007108 PMC390322

[B27] Chan S Bao Y Ciszak E Laget S Xu S Catalytic domain of restriction endonuclease BmrI as a cleavage module for engineering endonucleases with novel substrate specificities Nucleic Acids Res 2007 35 18 6238 6248 10.1093/nar/gkm665 17855396 PMC2094064

[B28] Maeder ML Thibodeau-Beganny S Osiak A Wright DA Anthony RM Eichtinger M Jiang T Foley JE Winfrey RJ Townsend JA Unger-Wallace E Sander JD Müller-Lerch F Fu F Pearlberg J Göbel C Dassie JP Pruett-Miller SM Porteus MH Sgroi DC Iafrate AJ Dobbs D McCray PB Jr Cathomen T Voytas DF Joung JK Rapid 'open-source' engineering of customized zinc-finger nucleases for highly efficient gene modification Mol Cell 2008 31 2 294 301 10.1016/j.molcel.2008.06.016 18657511 PMC2535758

[B29] Wu J Kandavelou K Chandrasegaran S Custom-designed zinc finger nucleases: what is next? Cell Mol Life Sci 2007 64 22 2933 2944 10.1007/s00018-007-7206-8 17763826 PMC2921987

[B30] Carroll D Progress and prospects: zinc-finger nucleases as gene therapy agents Gene Ther 2008 15 22 1463 1468 10.1038/gt.2008.145 18784746 PMC2747807

[B31] Nakajima A Tauchi T Sashida G Sumi M Abe K Yamamoto K Ohyashiki J Ohyashiki K Telomerase inhibition enhances apoptosis in human acute leukemia cells: possibility of antitelomerase therapy Leukemia 2003 17 3 560 567 10.1038/sj.leu.2402825 12646945

[B32] Guo Z Li Q Bartlett D Yang J Fang B Gene transfer: the challenge of regulated gene expression Trends Mol Med 2008 14 9 410 418 10.1016/j.molmed.2008.07.003 18692441

[B33] Yu EY Kim SE Kim JH Ko JH Cho MH Chung IK Sequence-specific DNA recognition by the Myb-like domain of plant telomeric protein RTBP1 J Biol Chem 2000 275 31 24208 24214 10.1074/jbc.M003250200 10811811

[B34] Hardy S Kitamura M Harris-Stansil T Dai Y Phipps M Construction of adenovirus vectors through Cre-lox recombination J Virol 1997 71 3 1842 1849 9032314 10.1128/jvi.71.3.1842-1849.1997 PMC191254

[B35] Carpenter AE Jones TR Lamprecht MR Clarke C Kang IH Friman O Guertin DA Chang JH Lindquist RA Moffat J Golland P Sabatini DM CellProfiler: image analysis software for identifying and quantifying cell phenotypes Genome Biol 2006 7 10 R100 10.1186/gb-2006-7-10-r100 17076895 PMC1794559

[B36] Dimri G Lee X Basile G Acosta M Scott G Roskelley C Medrano E Linskens M Rubelj I Pereira-Smith O A biomarker that identifies senescent human cells in culture and in aging skin *in vivo* Proc Natl Acad Sci USA 1995 92 20 9363 9367 10.1073/pnas.92.20.9363 7568133 PMC40985

