# Supplementary figures and images for: Creation of a novel telomere-cutting endonuclease based on the EN domain of telomere-specific non-long terminal repeat retrotransposon, TRAS1
Source: Mob DNA. 2010 Apr 1;1:13. doi: 10.1186/1759-8753-1-13 (PMC2868851; doi:10.1186/1759-8753-1-13)

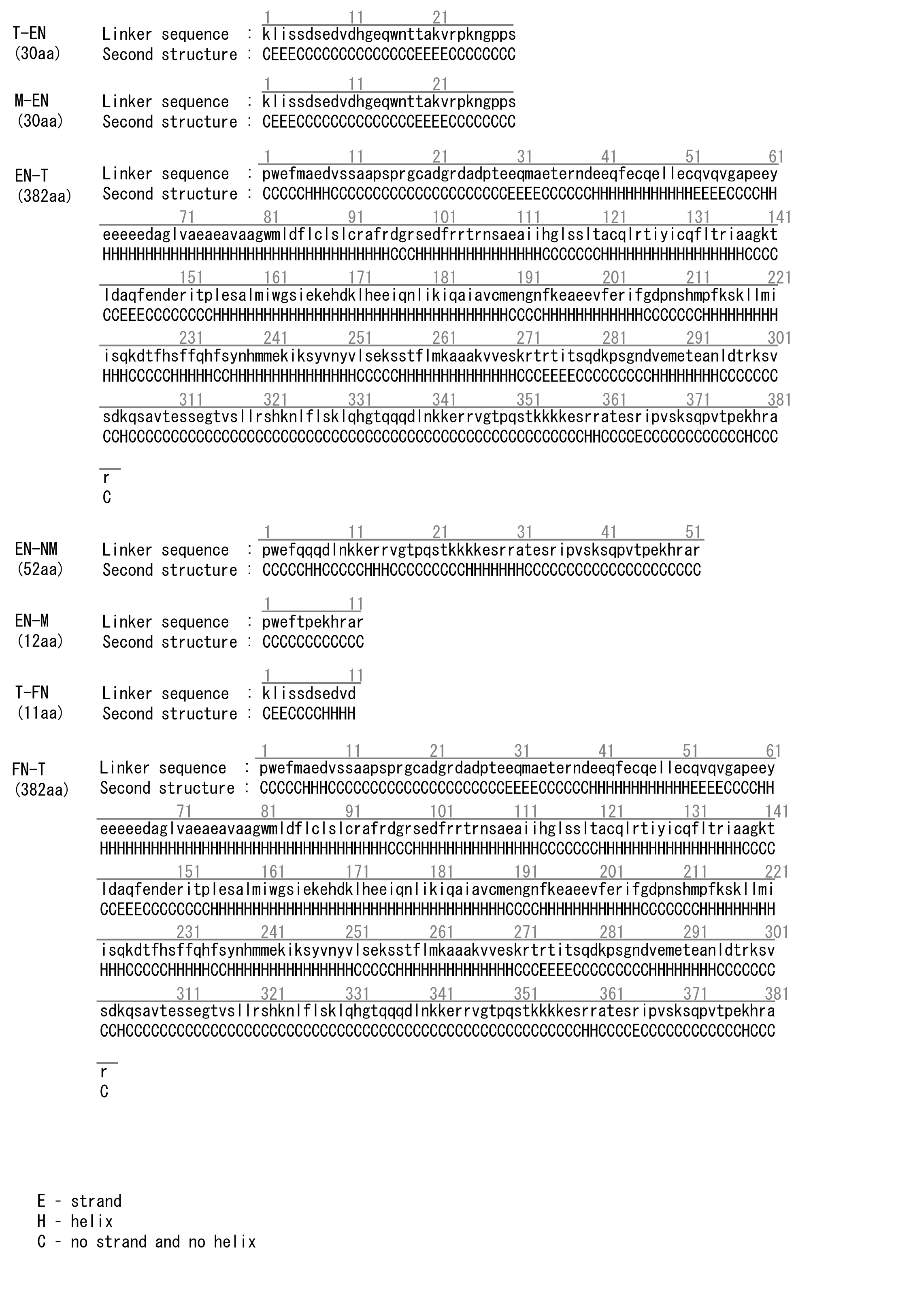

Supplement: Additional file 1 — Figure S1 - The amino acid sequences of protein linkers. The protein linker sequences of T-EN, M-EN, EN-T, EN-NM, EN-M, T-FN and FN-T are shown. The second structures of these proteins are predicted by SSpro http://scratch.proteomics.ics.uci.edu/. 67 -267 a.a. of the linker of EN-T and FN-T are TRF Homology domain (TRFH) which has tight structure, so we calculated linker length except this domain. Most of these linkers are nonstructural peptide. [file 1759-8753-1-13-S1.JPEG]

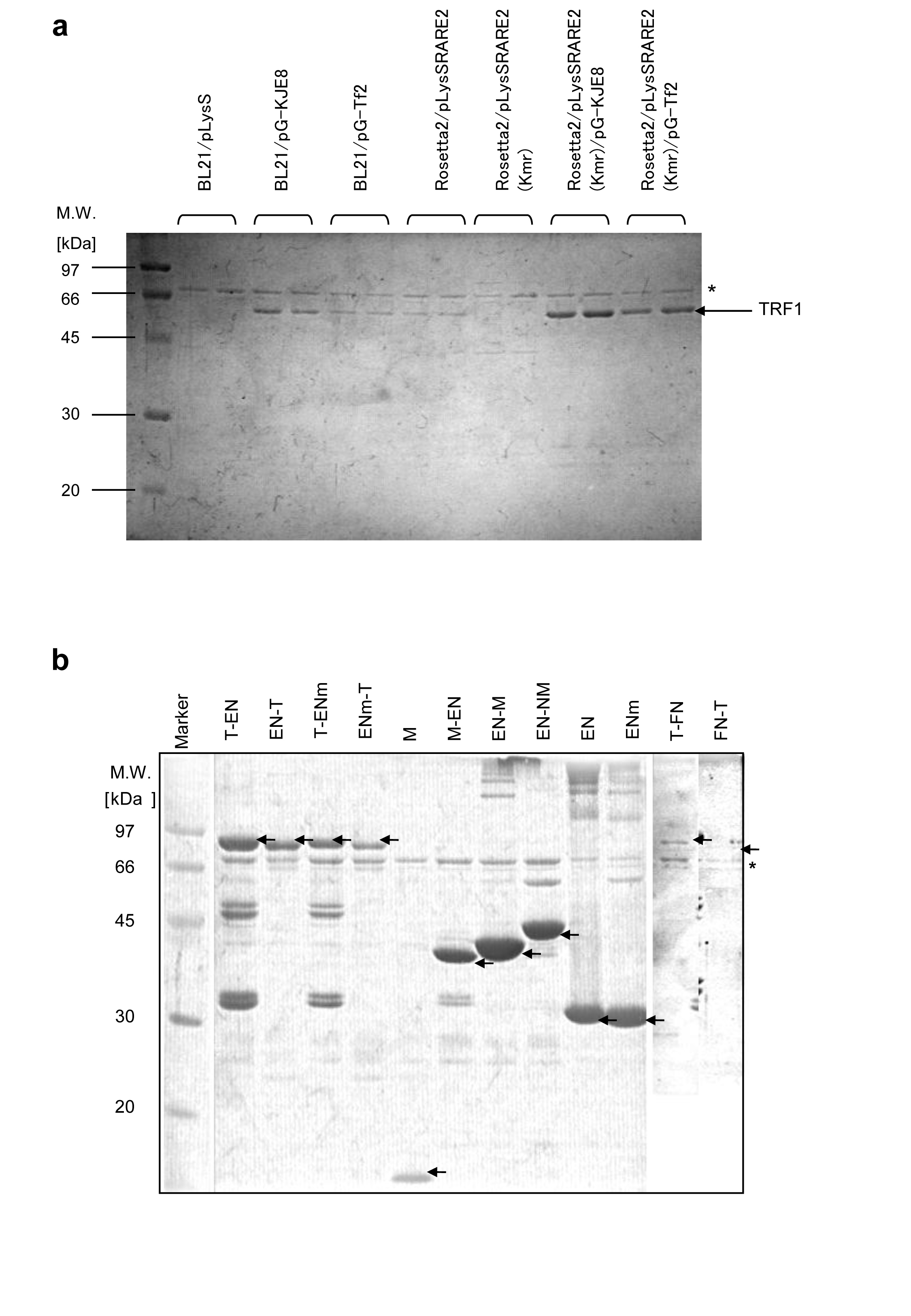

Supplement: Additional file 2 — Figure S2 - SDS-PAGE of purified chimeric endonucleases. (a) Selection of an appropriate E. coli strain for the effective expression of human protein (TRF1). Recombinant TRF1 protein was purified with a His tag. Protein extracts produced from bacterial cells were purified using Ni-NTA (nitrilotriacetic acid) agarose beads, and separated on a sodium dodecyl sulphate (SDS)-PAGE gel and stained with Coomassie brilliant blue. We confirmed that the molecular mass of overexpressed protein is identical to TRF1 (53.6 kDa). * indicates non-specific protein bound to Ni-NTA beads. The expression of human protein TRF1 increased with chaperon and rare tRNA. (b) SDS-PAGE of purified chimeric endonucleases using Rossetta2/pLysSRARE2 (Kmr) strain in (a). The molecular weight of each purified protein was the same as the calculated weight. Arrows shows purified protein. [file 1759-8753-1-13-S2.JPEG]

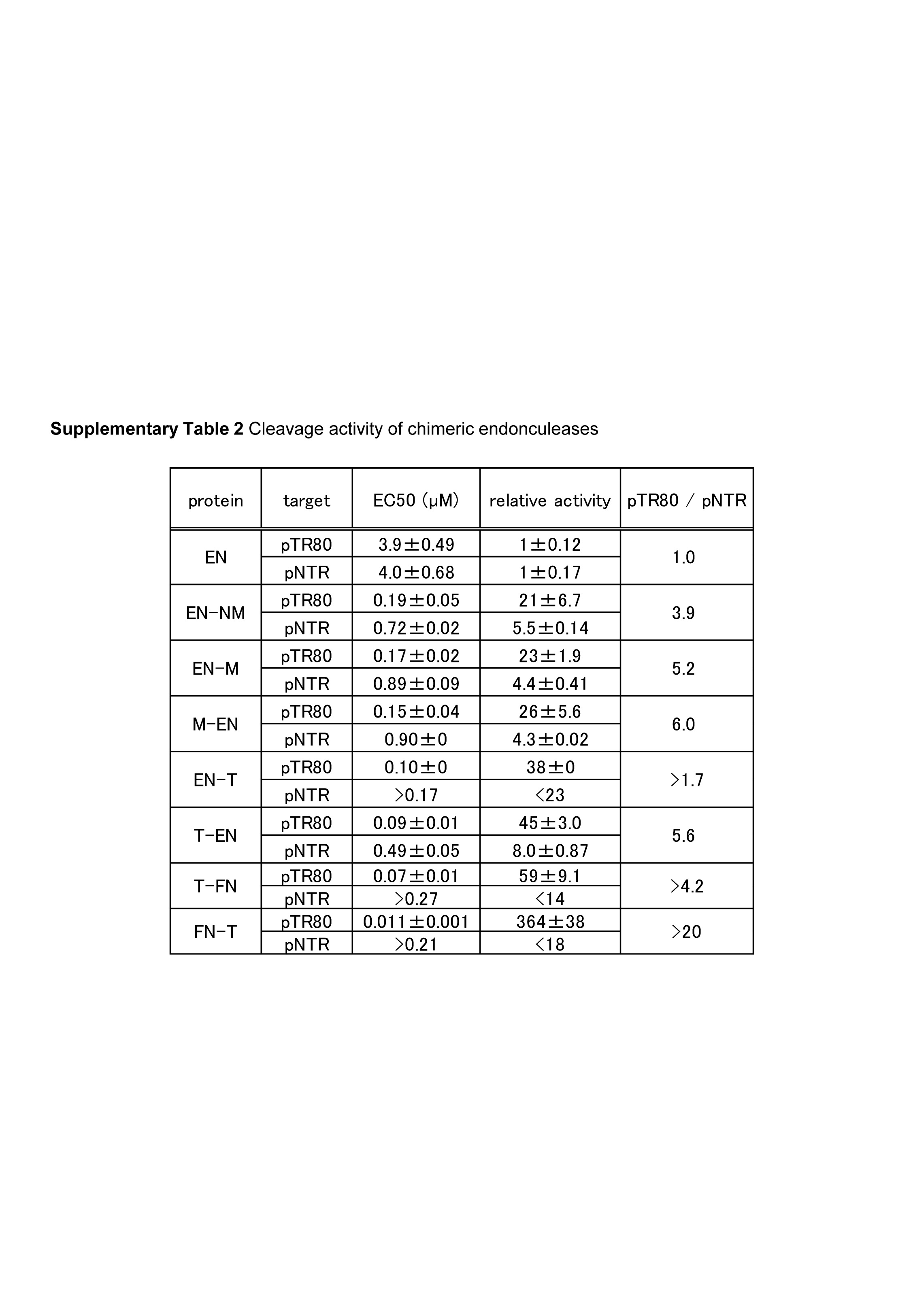

Supplement: Additional file 3 — Table S1. Cleavage activity of chimeric endonucleases. [file 1759-8753-1-13-S3.JPEG]

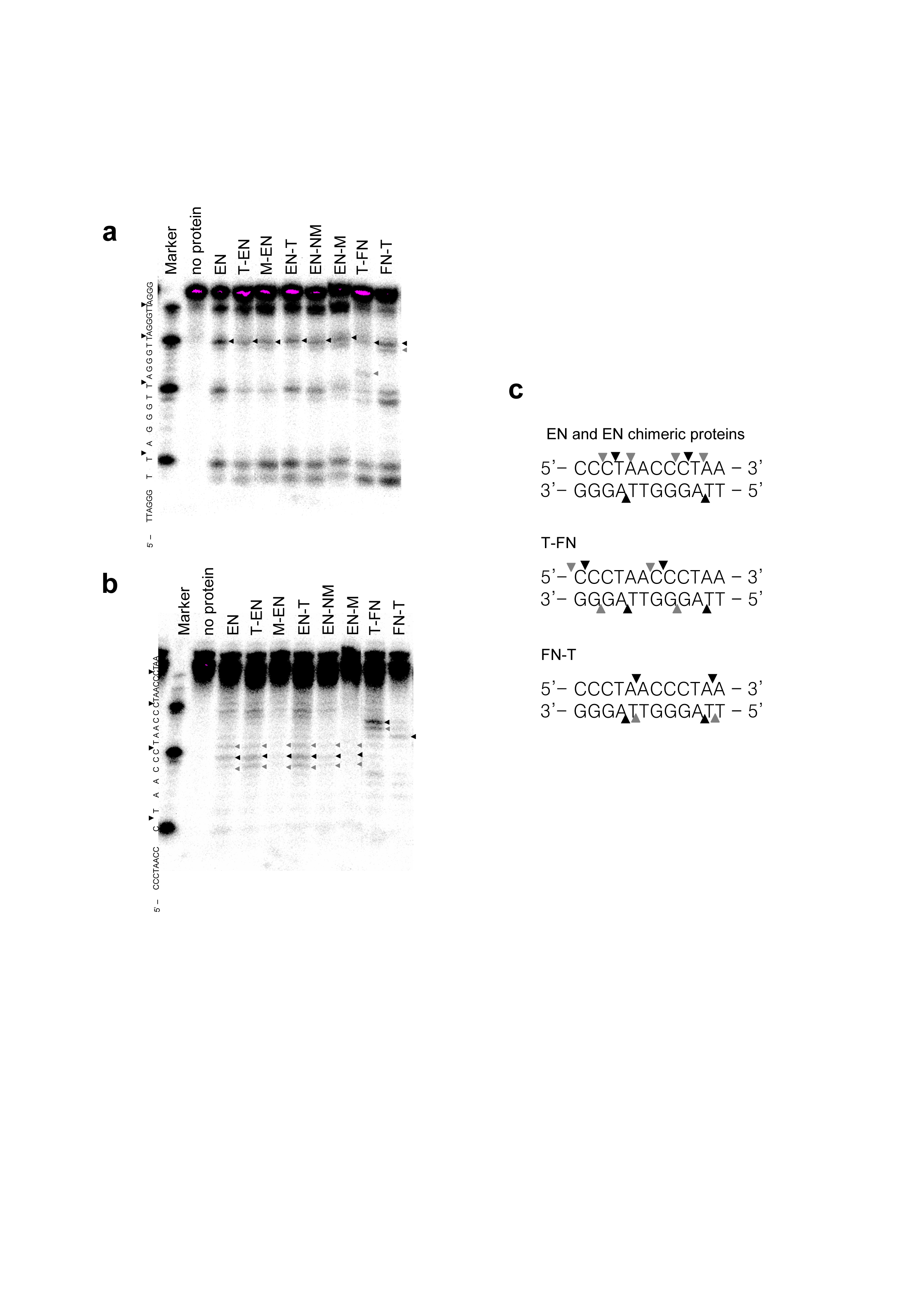

Supplement: Additional file 4 — Figure S3 - Cleavage of telomeric repeats by chimeric endonucleases. (a, b) RI-labelled double-strand oligo DNA substrate, TR5 containing 5 TTAGGG repeats was digested with chimeric endonuclease, and separated on polyacrylamide gels. The DNA concentration was 100 nM, and protein concentration was about 0.3 μM except for EN (6 μM), and FN-T (0.02 μM). The (TTAGGG)5 strand of TR5 was labelled in (a) and the (CCCTAA)5 strand was labelled in (b). Black arrowheads show major cleavage bands which were cleaved in every telomeric repeat; gray arrowheads show minor cleavage bands. (c) Schematic cleavage sites are shown based on the results of (a) and (b). [file 1759-8753-1-13-S4.JPEG]

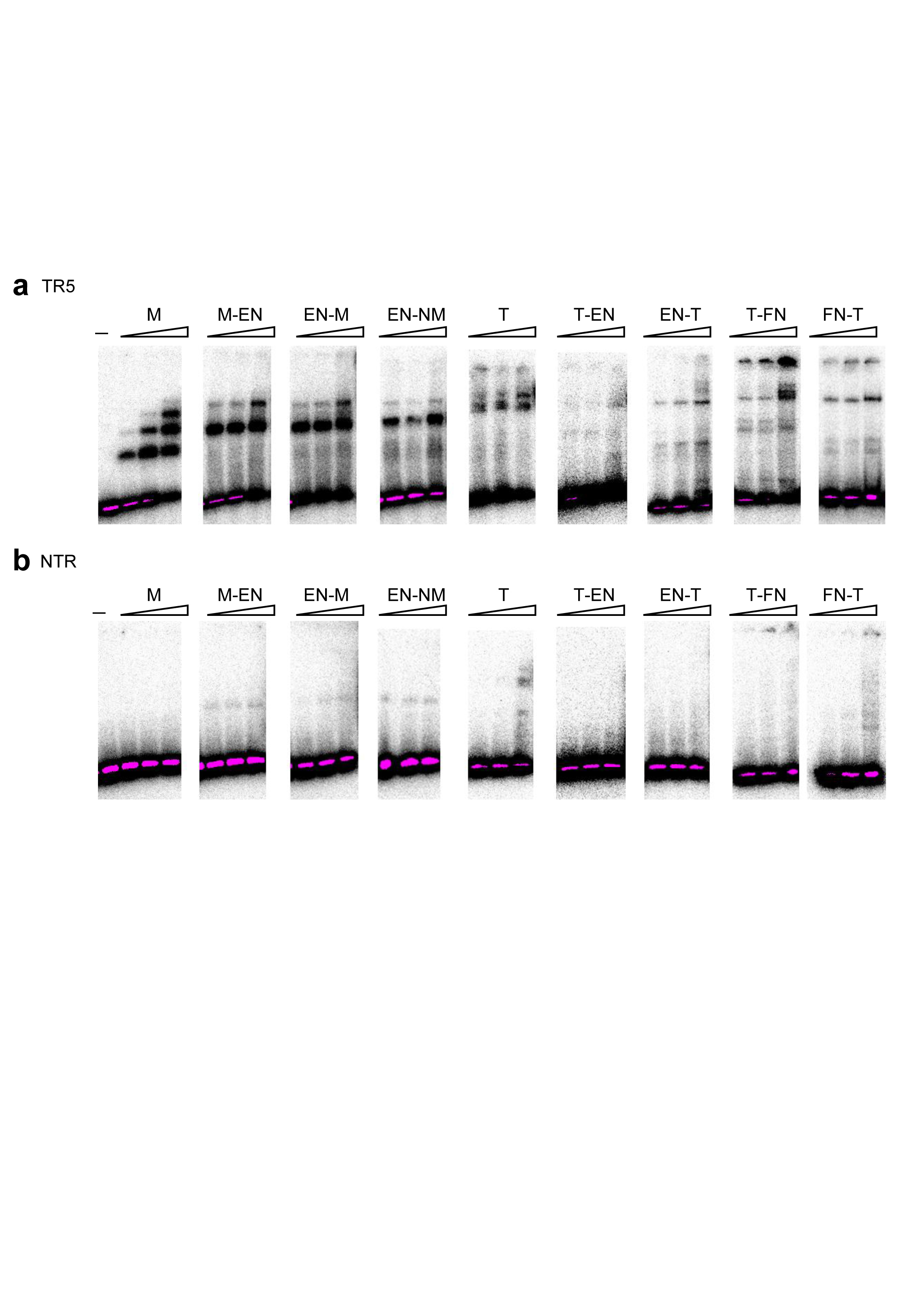

Supplement: Additional file 5 — Figure S4 - Binding activity of chimeric endonucleases to telomeric repeats. In order to examine the binding capacity of chimeric endonuclease to the telomeric repeats, we performed electrophoretic mobility shift assays. Approximately 1 nM RI-labelled double-strand oligo DNA was added with fusion protein at 0.04, 0.2, 1 μM except for T-FN (0.02, 0.1, 0.5 μM) and FN-T (0.01, 0.07, 0.3 μM) from left to right in each gel. Target oligo DNA used as probe was TR5, (TTAGGG)5 in (a), and NTR in (b). [file 1759-8753-1-13-S5.JPEG]

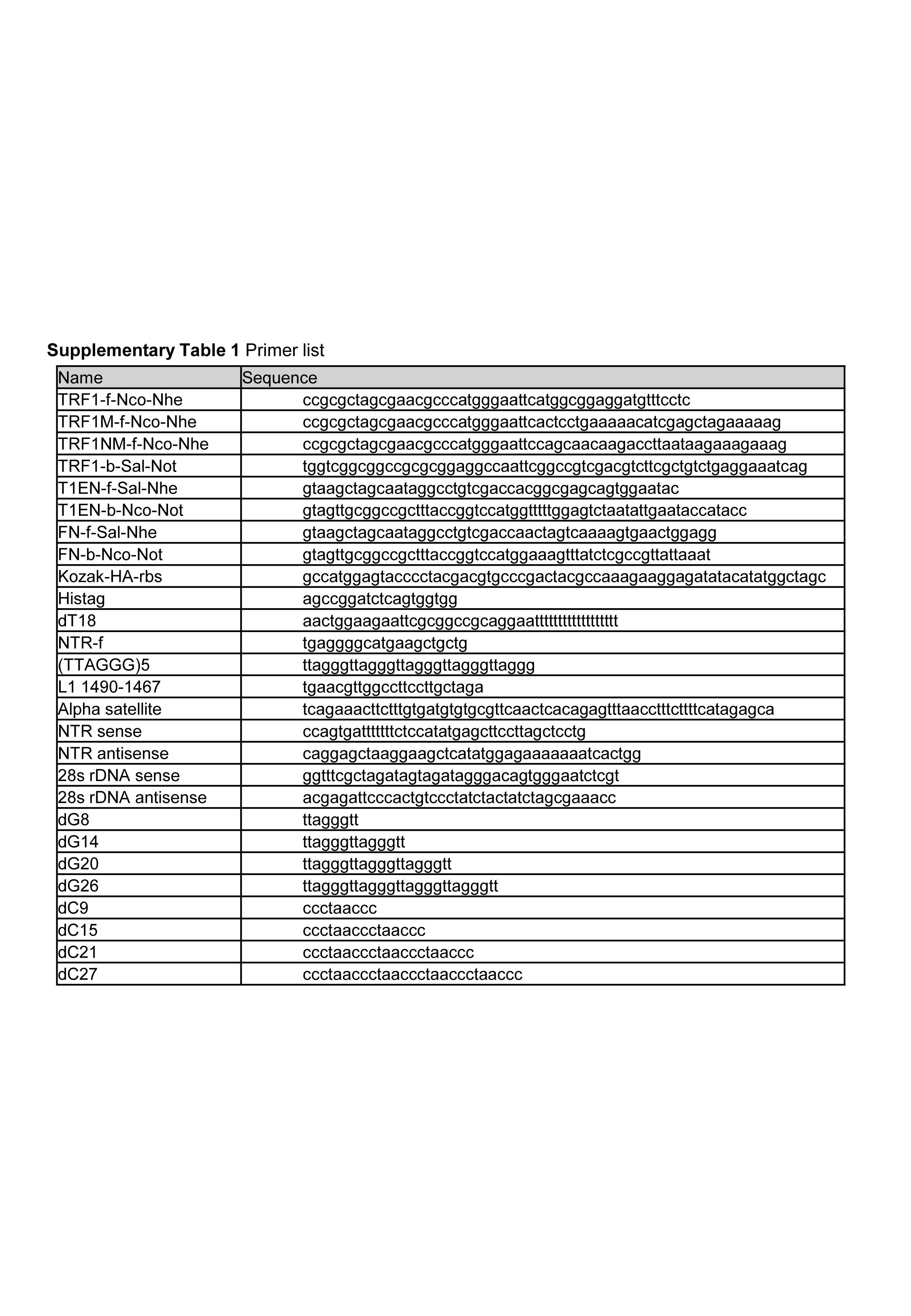

Supplement: Additional file 6 — Table S2. Primer list. [file 1759-8753-1-13-S6.JPEG]

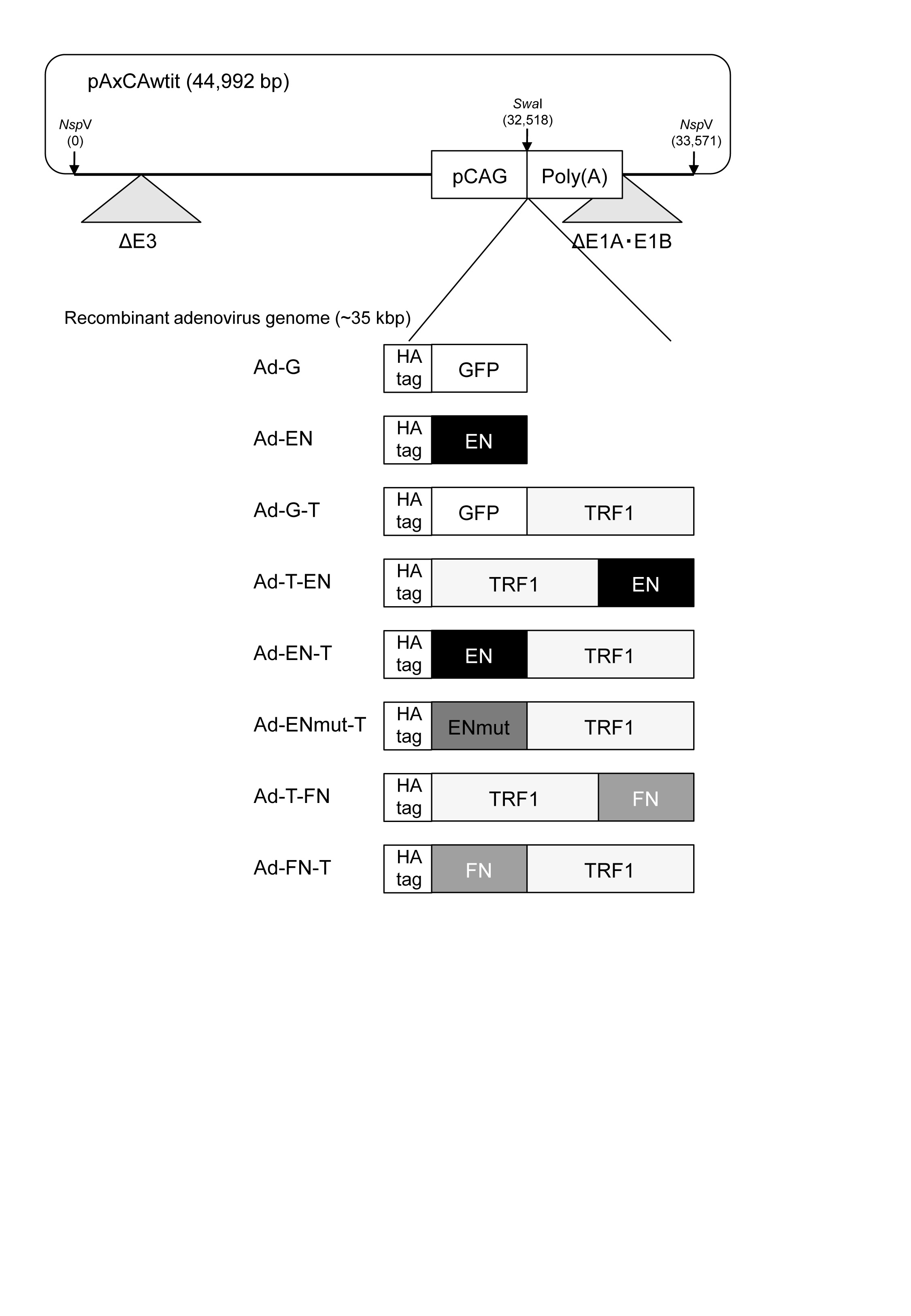

Supplement: Additional file 7 — Figure S5 - The map of recombinant adenovirus genome. The maps of recombinant adenoviruses, Ad-G, Ad-EN, Ad-GT, Ad-T-EN, Ad-EN-T, Ad-ENmut-T, Ad-T-FN and Ad-FN-T are shown. pAxCAwtit vector contains adenovirus genome between NspV sites. Adenovirus vectors are replication defective by deletion of the E1A and E1B genes which are required for virus replication in mammalian cells, and lack E3 gene which is not essential for virus replication in 293 cells. Recombinant proteins have HA epitope tag at the N-terminal end. [file 1759-8753-1-13-S7.JPEG]
